# Disentangling isolated dental remains of Asian Pleistocene hominins and pongines

**DOI:** 10.1371/journal.pone.0204737

**Published:** 2018-11-01

**Authors:** Tanya M. Smith, Alexandra Houssaye, Ottmar Kullmer, Adeline Le Cabec, Anthony J. Olejniczak, Friedemann Schrenk, John de Vos, Paul Tafforeau

**Affiliations:** 1 Australian Research Centre for Human Evolution, Griffith University, Nathan, Queensland, Australia; 2 Department of Human Evolutionary Biology, Harvard University, Cambridge, Massachusetts, United States of America; 3 ESRF—The European Synchrotron, Grenoble, France; 4 Département Adaptations du Vivant, UMR 7179 CNRS, Muséum National d’Histoire Naturelle, Paris, France; 5 Department of Paleoanthropology, Senckenberg Research Institute and Natural History Museum Frankfurt, Frankfurt a.M., Germany; 6 Department of Paleobiology and Environment, Institute of Ecology, Evolution, and Diversity, Johann Wolfgang Goethe University, Frankfurt a.M., Germany; 7 Department of Human Evolution, Max Planck Institute for Evolutionary Anthropology, Leipzig, Germany; 8 Independent Scientist, Las Vegas, Nevada, United States of America; 9 Department of Geology, Naturalis Biodiversity Center, Leiden, The Netherlands; Monash University, AUSTRALIA

## Abstract

Scholars have debated the taxonomic identity of isolated primate teeth from the Asian Pleistocene for over a century, which is complicated by morphological and metric convergence between orangutan (*Pongo*) and hominin (*Homo*) molariform teeth. Like *Homo erectus*, *Pongo* once showed considerable dental variation and a wide distribution throughout mainland and insular Asia. In order to clarify the utility of isolated dental remains to document the presence of hominins during Asian prehistory, we examined enamel thickness, enamel-dentine junction shape, and crown development in 33 molars from G. H. R. von Koenigswald's Chinese Apothecary collection (11 *Sinanthropus officinalis* [= *Homo erectus*], 21 “*Hemanthropus peii*,*”* and 1 “*Hemanthropus peii”* or *Pongo*) and 7 molars from Sangiran dome (either *Homo erectus* or *Pongo)*. All fossil teeth were imaged with non-destructive conventional and/or synchrotron micro-computed tomography. These were compared to *H*. *erectus* teeth from Zhoukoudian, Sangiran and Trinil, and a large comparative sample of fossil *Pongo*, recent *Pongo*, and recent human teeth. We find that *Homo* and *Pongo* molars overlap substantially in relative enamel thickness; molar enamel-dentine junction shape is more distinctive, with *Pongo* showing relatively shorter dentine horns and wider crowns than *Homo*. Long-period line periodicity values are significantly greater in *Pongo* than in *H*. *erectus*, leading to longer crown formation times in the former. Most of the sample originally assigned to *S*. *officinalis* and *H*. *erectus* shows greater affinity to *Pongo* than to the hominin comparative sample. Moreover, enamel thickness, enamel-dentine junction shape, and a long-period line periodicity value in the “*Hemanthropus peii*” sample are indistinguishable from fossil *Pongo*. These results underscore the need for additional recovery and study of associated dentitions prior to erecting new taxa from isolated teeth.

## Introduction

Fossil remains attributed to *Homo erectus* have been recovered from numerous localities in mainland Asia and island Southeast Asia driven, in part, by intense scholarly interest during the last century in a potential Asian origin for the genus *Homo*. These recoveries derive from more than a dozen sites with well-preserved hominin cranial material (reviewed in [1–3]). More problematic are localities where only isolated teeth or fragmentary jaws have been discovered (e.g., Longgupo, China: [4]; most Vietnamese localities) (Table A in S1 File), as *H*. *erectus* likely coexisted with large-bodied apes [5, 6]. Morphological and metrical convergence between Asian hominin and pongine post-canine dental material has confounded paleoanthropologists for decades [5, 7–12]. This is exemplified by the phenomenon of variably wrinkled (crenulated) molar occlusal surfaces with low cuspal relief.

Taxonomic assessments of mixed primate faunal collections, such as those from Chinese Apothecaries [13, 14], the Sangiran dome [5, 15], and Mohui Cave [12, 16–18] have yielded varied opinions about the affinities of these isolated dental remains. An example is von Koenigswald's identification of a new hominin “*Hemanthropus peii*” from a subset of his Chinese Apothecary collection [14, 19], which has since been considered to be an australopithecine ([20, 21], but see [22, 23]), a member of the genus *Homo* [24], an assortment of fossil orangutans [9, 12, 25], and/or a new type of hominoid [9, 12, 18]. Von Koenigswald [14] originally reported that this taxon was “difficult to define” and distinguished several isolated molar teeth from orangutans solely on the lack of “fine wrinkles” and resulting “better defined cusps,” promising a more detailed description that was never published. Yet both orangutans and humans show marked intraspecific variation in the expression of crenulations and the underlying cuspal morphology [7, 26]. Moreover crenulations are compromised by wear, making this a tenuous morphological feature on which to erect a new genus and species.

Tooth enamel thickness has been used to assess the taxonomic affiliations of primate dental remains for the past century [27–33]. Enamel-dentine junction shape has also proven to distinguish primate groups at family, genus, and species levels [30, 34–38]. Aspects of dental development, most notably the intrinsic long-period line repeat interval, daily secretion rate, and tooth crown formation time, also differ among some hominin and hominoid taxa (reviewed in [39, 40]).

This study quantifies aspects of internal tooth structure and development using non-destructive imaging to examine the taxonomic affiliation of isolated teeth from the Asian Pleistocene. Recent studies have explored aspects of tooth structure and development with propagation phase contrast synchrotron micro-computed tomography (SR microCT) and conventional microCT for better resolution of Pleistocene Asian primates than is possible from traditional assessments of tooth size and shape [30, 33, 36, 41–43]. We have expanded this approach in the current study by considering a suite of dental characteristics in two historic collections of teeth, which are compared to a large sample of recent and fossil orangutans and hominins.

## Materials and methods

### Sample

We examined two-dimensional (2D) relative enamel thickness, enamel-dentine junction shape, and crown development in 33 molars from von Koenigswald's Chinese Apothecary (CA) collection (11 *Sinanthropus officinalis* [= *Homo erectus*], 21 “*Hemanthropus peii*,*”* 1 “*Hemanthropus peii”* or *Pongo*) and 7 teeth from Sangiran dome (either *H*. *erectus* or *Pongo)* housed at the Senckenberg Research Institute (Frankfurt, Germany) (Table 1) (Figs A-C in S1 File) [5, 13, 14, 19, 44]. All teeth were imaged with conventional and/or synchrotron microCT as detailed below. High-resolution surface impressions and stereomicrographs were also prepared following established procedures [30]. These fossil molars were compared to ten securely identified *H*. *erectus* teeth from Sangiran, Zhoukoudian, and Trinil [5, 30, 42, 45–52], more than 160 fossil *Pongo* teeth [53, 54], and a large sample of recent human and orangutan molars [54–57]. Novel data were collected during this study for a subset of the comparative sample, including developmental data from four *H*. *erectus* teeth from Zhoukoudian housed at the Museum of Evolution (Uppsala University) and five fossil *Pongo* molars housed at the Senckenberg Research Institute (Frankfurt, Germany) and the Naturalis Biodiversity Center (Leiden). No permits were required for the described study, which complied with all relevant regulations.

**Table 1:** Pleistocene dental remains included in this study.

Collection	Accession	Tooth	Original Attribution	Study Method
*Ungrouped Cases*				
Chinese	673	RUM3	*Hemanthropus peii* (type)	MicroCT & SR MicroCT
Apothecary	674	RLM1-2	*Hemanthropus peii*	MicroCT
	675	RLM1-2	*Hemanthropus peii*	MicroCT
	677	RLM1-2	*Hemanthropus peii*	MicroCT
	678	RLM1-2	*Hemanthropus peii*	MicroCT
	685	LLM1-2	*Hemanthropus peii*!?	MicroCT
	686	LLM1-2	*Hemanthropus peii*	MicroCT
	689	LLM1-2	*Hemanthropus peii*	MicroCT
	690	RUM1-2	*Hemanthropus peii*	MicroCT
	692	LUM1-2	*Hemanthropus peii*	MicroCT
	695	LUM1-2	*Hemanthropus peii*	MicroCT
	696	LUM1-2	*Hemanthropus peii*	MicroCT
	703	RUM1-2	*Hemanthropus peii*	MicroCT
	704	RUM1-2	*Hemanthropus peii*	MicroCT
	705	RUM1-2	*Hemanthropus peii*	MicroCT
	707	LUM1-2	*Hemanthropus peii*	MicroCT
	709	LUM1-2	*Hemanthropus peii*	MicroCT
	724	RUM1-2	*Hemanthropus peii*	MicroCT
	725	RUM1-2	*Hemanthropus peii*	MicroCT
	728	LLM3	*Hemanthropus peii*	MicroCT
	729	LLM1-2	*Hemanthropus peii*	MicroCT
	730	LLM1-2	*Hemanthropus peii* or *Pongo*?	MicroCT
	770	RUM1	*Sinanthropus officinalis* (type)	MicroCT & SR MicroCT
	771	LUM1-2	*Sinanthropus officinalis*	MicroCT & SR MicroCT
	772	LUM1-2	*Sinanthropus officinalis*	MicroCT & SR MicroCT
	796	RUM1-2	*Sinanthropus officinalis*	MicroCT & SR MicroCT
	799	RUM1-2	*Sinanthropus officinalis*	MicroCT
	804	LLM1-2	*Sinanthropus officinalis*	MicroCT & SR MicroCT
	805	RLM1-2	*Sinanthropus officinalis*	MicroCT & SR MicroCT
	806	LLM1-2	*Sinanthropus officinalis*	MicroCT & SR MicroCT
	807	RLM1-2	*Sinanthropus officinalis*	MicroCT & SR MicroCT
	808	RLM3	*Sinanthropus officinalis*	MicroCT & SR MicroCT
	816	RLM3	*Sinanthropus officinalis*	MicroCT & SR MicroCT
Sangiran	S7-9	RUM1	*Homo erectus*	MicroCT & SR MicroCT
	S7-20	LLM1-2	*probably fossil Pongo*	MicroCT & SR MicroCT
	S7-53	LUM2	*Homo erectus*	MicroCT & SR MicroCT
	S7-62	RLM1-2	*probably fossil Pongo*	MicroCT & SR MicroCT
	S7-64	RLM2	*Homo erectus*	MicroCT & SR MicroCT
	S7-65	RLM2	*probably fossil Pongo*	MicroCT & SR MicroCT
	S7-76	RLM1	*Homo erectus*	MicroCT & SR MicroCT
*Comparative hominin samples*				
Zhoukoudian	M3549	LLP3	*Homo erectus*	SR MicroCT
	M3550	RUM3	*Homo erectus*	SR MicroCT
	M3887	RLP4	*Homo erectus*	SR MicroCT
	PMU 25719	RUC	*Homo erectus*	SR MicroCT
Trinil	11620	RUM3-4	*Homo erectus*	MicroCT & SR MicroCT
	11621	LUM2-3	*Homo erectus*	MicroCT & SR MicroCT
Sangiran	S4	RUM2	*Homo erectus*	MicroCT
	S4	RUM3	*Homo erectus*	MicroCT
	S7-37	RUP4	*Homo erectus*	Physical histology
	S7-37	RUM1	*Homo erectus*	Physical histology

Tooth: R—right, L—left, U—upper, L—lower, C—canine, P—premolar, M—molar. Original Attribution of Chinese Apothecary (CA) teeth from von Koenigswald (recorded in the Senckenberg Research Institute and Natural History Museum Frankfurt Catalogue), and Sangiran teeth from Grine [5]. Comparative *Homo erectus* material is discussed in [5, 30, 42, 45–52] as well as novel data collected on the Zhoukoudian teeth during this study.

### Imaging

We initially employed a Skyscan 1172 microCT housed in the Department of Human Evolution at the Max Planck Institute for Evolutionary Anthropology to scan 33 teeth from the Chinese Apothecary collections and 57 teeth from the Sangiran dome at 100 kV and 100 mA, with a metallic filter (0.04 mm of copper plus 0.5 mm of aluminum) and an isometric voxel size of 14–27 μm. Unfortunately, due to diagenetic modification [58, 59], it was not possible to virtually distinguish the interface between enamel and dentine in the great majority of the Sangiran dome dental sample.

Seven teeth from the Sangiran dome collection, along with 11 *Sinanthropus officinalis* (= *Homo erectus*) teeth and the “*Hemanthropus peii*” type (CA 673) from the Chinese Apothecary collection, were subsequently scanned on beamline ID 19 at the European Synchrotron Radiation Facility (ESRF, Grenoble, France) following the protocol detailed below. Four *H*. *erectus* teeth from Zhoukoudian and one Chinese fossil orangutan tooth (CA 260) were also similarly imaged as part of the comparative sample.

We used two optical configurations to reveal overall tooth crown structure and aspects of the microstructure. Scans of entire teeth were performed with a FReLoN 2K CCD camera coupled to a LuAG:Ce scintillator of 200 μm thick with an optical system yielding a pixel size of 4.96 μm. The beam was set at an average energy of 60.8 keV by filtering the white beam of the W150 wiggler set at a gap of 65 mm with 2 mm of aluminum and 0.25 mm of tungsten. We used a propagation distance of 4 m in order to reveal the general growth pattern and the enamel-dentine junction. The scans were performed in half-acquisition mode with 5000 projections of 0.3 seconds each over 360 degrees.

Most of the high-resolution propagation phase contrast scans used to investigate the long-period line periodicity were performed using a FReLoN E2V CCD camera coupled to a 10 μm thick GGG:Eu scintillator through a microscope optic yielding a pixel size of 0.6 μm. The beam was set at an average energy of 59.7 keV by filtering the white beam of the two U32 undulators closed respectively at 11.88 and 11.72 mm gaps with 2 mm of aluminum and 0.25 mm of tungsten. We used a propagation distance of 150 mm to reveal incremental lines with scans in half-acquisition mode using 5000 projections of 0.9 seconds each over 360 degrees. The *H*. *erectus* comparative sample was scanned using a different microscope optic adapted for white beam, producing a pixel size of 0.73 μm. In this case we used a FReLoN 2K14 CCD camera in frame transfer mode, a 24 μm thick GGG:eu scintillator, and 5000 projections of 0.3 seconds each in half-acquisition mode. The average energy of the beam (68.3 keV) was set by filtering the white beam of the ID19 W150 wiggler set at a gap of 35 mm by 3 mm of aluminum, 0.25 mm of copper and 0.06 mm of tungsten. A suitable phase contrast effect was obtained with a propagation distance of 150 mm.

For some teeth, the high resolution scans led to significant local darkening of the enamel. The samples were restored to their original color by illumination with low energy UV light (black light neon tubes) for a few hours [60]. Recent refinements subsequent to the acquisition of these data have reduced the radiation dose to avoid this darkening effect [61]. Volumes were reconstructed using filtered-backprojection algorithm (PyHST software, ESRF) in edge detection mode. Residual ring artefacts were corrected on reconstructed slices with a custom Matlab code [62]. The 3D renderings, virtual slices, and raw data employed in this study are available in the open access database for paleontology hosted by the ESRF (http://paleo.esrf.fr/picture.php?/3229/category/2223).

### Enamel structure analysis

**Fig 1:**
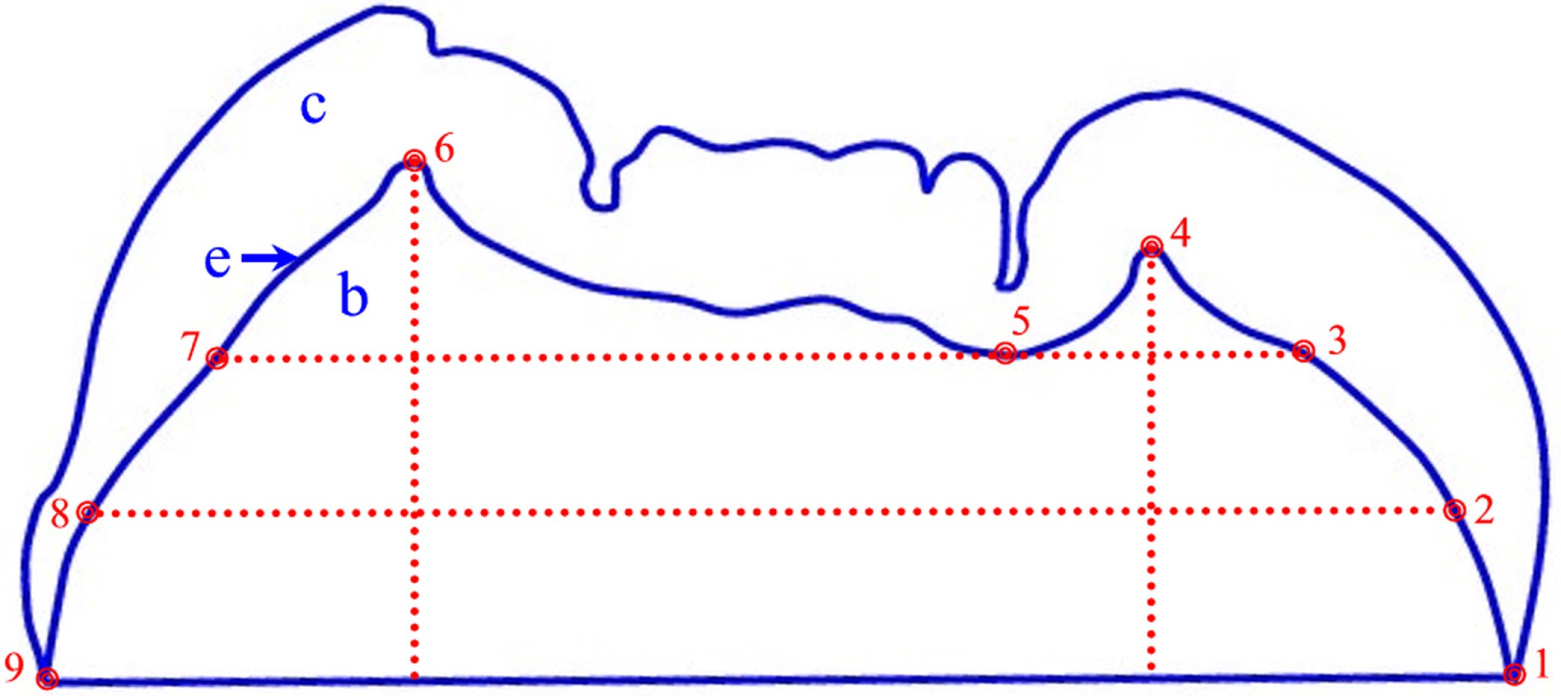
*Homo erectus* upper molar (Trinil 11620) tracing showing measurements for enamel thickness and enamel-dentine junction morphology characterization. The area of the enamel cap is represented as c, the area of the dentine under the enamel cap is represented as b, and the length of the enamel-dentine junction is represented as e. Landmarks are defined as follows: 1) tip of the lingual enamel cervix; 2) lingual intersection of the EDJ and a line parallel to the cervical diameter and bisecting the length between the cervical diameter and landmark 5; 3) lingual intersection of the EDJ and a line parallel to the cervical diameter and running through landmark 5; 4) mesiolingual dentine horn tip; 5) lowest point of the EDJ between the mesiolingual and mesiobuccal cusp tips; 6) mesiobuccal dentine horn tip; 7) buccal intersection of the EDJ and a line parallel to the cervical diameter and running through landmark 5; 8) buccal intersection of the EDJ and a line parallel to the cervical diameter and bisecting the length between the cervical diameter and landmark 5; and, 9) tip of the buccal enamel cervix. Landmark 1 was made to lie at x, y coordinate (0, 0) and landmark 9 at (0, 100) in every specimen examined in order to account for differences in tooth size. Reproduced from [30].

Due to diagenesis and wear of the molars it was not possible to conduct three-dimensional (3D) measurements of enamel thickness [63–65], nor 3D enamel-dentine junction (EDJ) morphology [33, 37, 64, 66], so a cross-sectional two-dimensional (2D) approach was taken. Virtual 2D section planes of the mesial cusps were generated from 3D models with VG Studio MAX 2.0 software (Volume Graphics, Inc.) according to published protocols [36, 57, 65, 67]. Several variables were quantified on 2D section planes with Sigma Scan Pro software interfaced to a Wacom digitizing tablet following Martin [28, 68]: enamel cap area (c), EDJ length (e), and coronal dentine area enclosed by the enamel cap (b) (Fig 1). Average enamel thickness (AET) was calculated as [c/e], yielding the average straight-line distance (in mm), or thickness, from the enamel-dentine junction to the outer enamel surface. AET was scaled for comparisons between taxa of different size by calculation of relative enamel thickness (RET; unitless index): [100 * [c/e]/ sq. rt. b]. Comparative molar enamel thickness data for recent *Pongo* (n = 135), fossil *Pongo* (n = 139), *H*. *erectus* (n = 4), and recent human molars (n = 271) was taken from [53, 55, 57, 67].

Molar EDJ morphology was quantified by collecting nine landmarks and semi-landmarks from each intact and lightly worn cross-section [34, 35] (Fig 1), and calculating a series of nine relative distances from these landmarks. These distances were combined with a database of homologous measurements of five *H*. *erectus* molars, 90 recent orangutans, 141 fossil orangutans, and 258 recent humans [30, 35, 53, 55, 57]. The relative distances were subjected to discriminant function analysis (DFA) using SPSS software (v. 21.0, IBM, Inc.). Variation in mesial cross-section EDJ shape from first to third molars is minimal and does not overwhelm the ability of this technique to distinguish taxa [35]. Moreover, as a number of these molars are of uncertain position within the dental arcade, molars from all three maxillary or mandibular positions were combined in the analyses. Sixteen putative hominin or pongine molars from the Chinese Apothecary and Sangiran dome collections were treated as ungrouped, or non-classified, cases in the DFA, and the sample size of each taxon was not used to adjust the probability of molars belonging to any group. A separate DFA of molar EDJ shape in 19 "*Hemanthropus peii*" molars was run, which also included specimens CA 770 and 771 in the *H*. *erectus* sample since their maxillary molar EDJ shapes and long-period line periodicity values strongly support their hominin affinities [30].

### Dental development analysis

**Fig 2:**
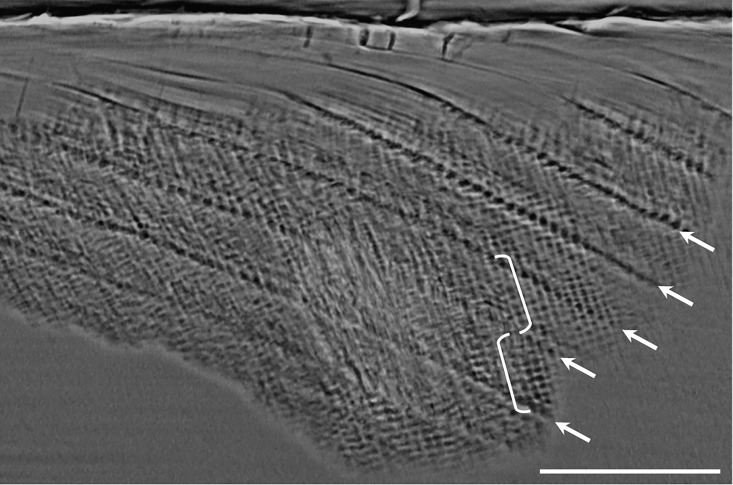
Virtual histological slice showing long-period line periodicity. Ten daily cross-striations can be seen (light and dark bands in white brackets) between long-period lines (arrows) in CA 808. The scale bar is equal to 100 μm.

Casts and photographs of the fossil sample revealed that very few of the tooth surfaces were sufficiently preserved for developmental assessment due to extreme postmortem pitting, etching and/or extensive tooth wear prior to death. Phase contrast SR microCT was employed to assess long-period line periodicity and crown formation times from virtual histological slices and 3D models of the original material (illustrated in [69]: Fig 1; [70]: Fig 8). Virtual histological slices (typically 30–100 μm thick) were produced following 3D optimized orientation to capture the full growth axis (dentine and pulpal horn tips). The periodicity, or number of daily lines between successive long-period incremental features (Fig 2), was examined in 23 fossil teeth. The Mann-Whitney U test was performed for comparisons of periodicity values between *H*. *erectus* and fossil *Pongo* teeth in the comparative sample.

Crown formation time was calculated for the few teeth that were sufficiently preserved as the sum of the cuspal and lateral enamel formation times, save for one instance when a complete series of long-period lines was identified and counted from the coronal dentine and then multiplied by the long-period line periodicity (CA 796). Cuspal formation time was estimated by dividing the linear enamel thickness of each tooth cusp by 4.2 μm/day, which is the average cuspal daily secretion rate for fossil *Pongo* (4.19 μm/day, n = 9 teeth: [54]) and *H*. *erectus* (4.23 μm/day, n = 1–2 teeth: [40]). Direct measurements of cuspal daily secretion rates were not made due to the time- and data-intensive nature of quantifying cross-striation spacing virtually in the full thickness of cuspal enamel.

Lateral enamel formation time was calculated by multiplying the number of enamel long-period lines (internal Retzius lines or external perikymata) by the long-period line periodicity (or periodicity range when a single integer was not discernible). Minor estimates of long-period line numbers were made for worn or chipped enamel; specimens were excluded when these could not be counted for at least 90% of the total crown height. Comparative data on long-period line periodicity and crown formation times were chosen to minimize potential bias from methodological differences or interobserver error; data sources are provided in corresponding tables and figures. Because crown formation times vary among molar cusps and between upper and lower molars [56], comparisons among samples and taxa were limited to specific cusp types.

## Results

### Enamel thickness

**Fig 3:**
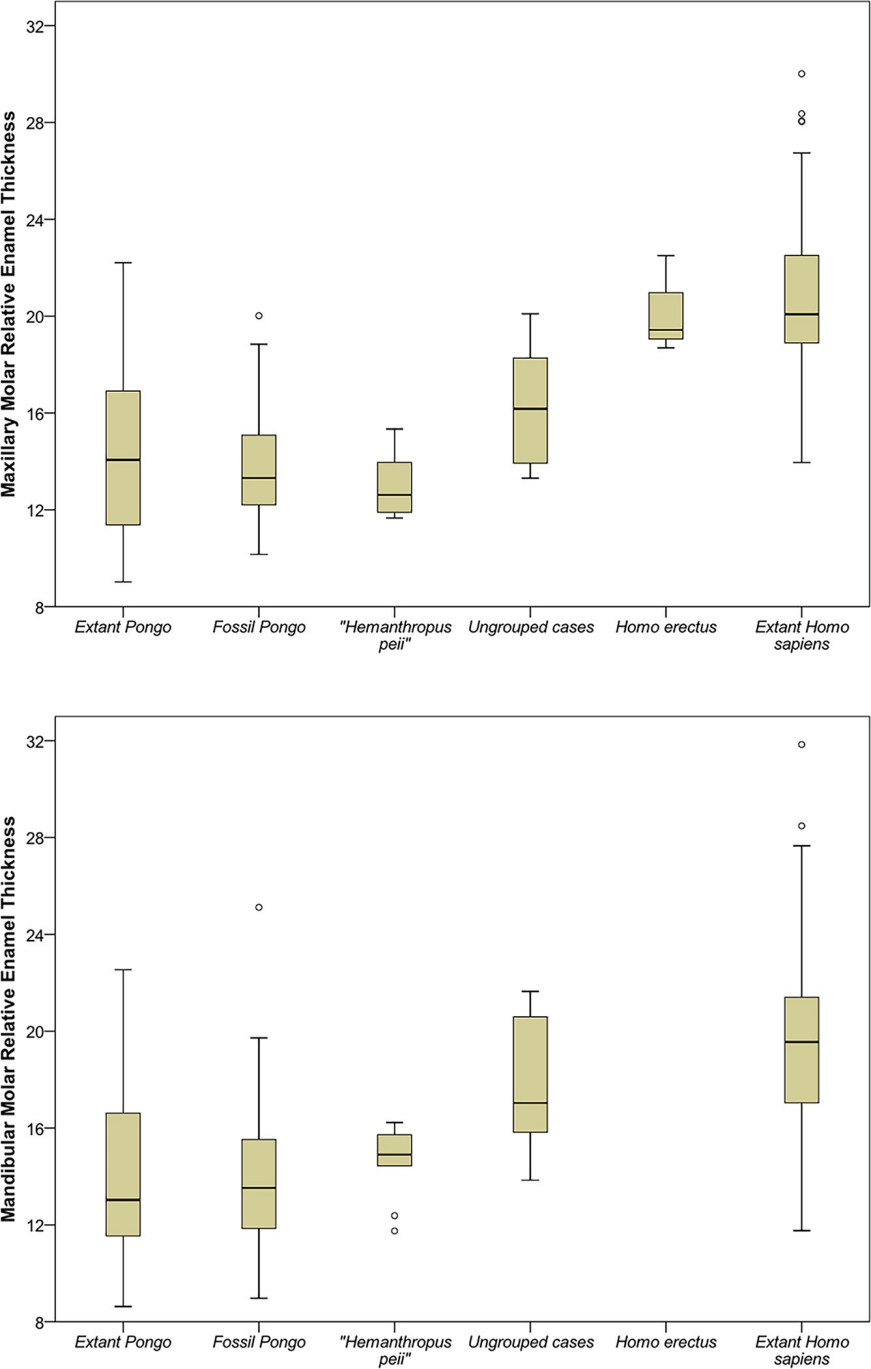
Two-dimensional relative enamel thickness values for maxillary (upper plot) and mandibular (lower plot) molars in the study sample (ungrouped cases) and comparative material. Standard box and whisker plot revealing the interquartile range (25th–75th percentiles: bars), 1.5 interquartile ranges (whiskers), and the median values (black line). Outliers are signified by circles. There are no data available regarding enamel thickness for mandibular molars of *H*. *erectus*.

Fig 3 reveals that 16 of the putative hominin or pongine molars (indicated as “ungrouped cases”) show a wide range of relative enamel thickness values that overlap with extant and fossil *Pongo* and *Homo*. The "*Hemanthropus peii*" sample (n = 19) shows the greatest similarity to fossil *Pongo* values, particularly for maxillary molars, with only a narrow overlap with *Homo*. The type specimen of "*Hemanthropus peii*" (CA 673) has a maxillary molar RET value of 13.1, which is similar to the fossil *Pongo* mean (13.8). Individual average and relative enamel thickness values are given in Table 2, along with range data for *H*. *erectus* and fossil *Pongo*.

**Table 2:** Two-dimensional molar average and relative enamel thickness values.

Collection	Accession	Tooth	AET (mm)	RET
*Ungrouped Cases*				
Chinese	673	RUM3	1.03	13.09
Apothecary	674	RLM1-2	1.23	14.75
	675	RLM1-2	1.24	14.44
	677	RLM1-2	1.34	15.72
	678	RLM1-2	1.27	16.23
	685	LLM1-2	1.13	14.91
	686	LLM1-2	n/a	n/a
	689	LLM1-2	1.10	14.90
	690	RUM1-2	1.07	12.35
	692	LUM1-2	0.92	11.75
	695	LUM1-2	0.90	11.66
	696	LUM1-2	1.06	11.97
	703	RUM1-2	0.99	11.90
	704	RUM1-2	1.16	15.34
	705	RUM1-2	0.99	12.88
	707	LUM1-2	1.19	15.27
	709	LUM1-2	n/a	n/a
	724	RUM1-2	n/a	n/a
	725	RUM1-2	1.19	13.96
	728	LLM3	1.51	16.14
	729	LLM1-2	1.01	11.75
	730	LLM1-2	0.91	12.38
	770	RUM1	1.34	18.27
	771	LUM1-2	1.19	15.61
	772	LUM1-2	n/a	n/a
	796	RUM1-2	1.17	13.93
	799	RUM1-2	0.87	13.31
	804	LLM1-2	1.40	21.48
	805	RLM1-2	0.95	13.85
	806	LLM1-2	1.16	15.17
	807	RLM1-2	n/a	n/a
	808	RLM3	1.74	20.59
	816	RLM3	1.15	16.38
Sangiran	S7-9	RUM1	1.15	16.73
	S7-20	LLM1-2	1.18	19.66
	S7-53	LUM2	1.42	20.10
	S7-62	RLM1-2	1.18	16.38
	S7-64	RLM2	1.28	17.69
	S7-65	RLM2	1.49	21.64
	S7-76	RLM1	1.13	15.82
*Comparative Fossil Sample*				
*H*. *erectus*	n = 4	maxillary	1.13–1.51	18.69–22.50
		mandibular	n/a	n/a
Fossil *Pongo*	n = 76	maxillary	0.76–1.42	12.83–20.02
	n = 63	mandibular	0.99–1.52	12.87–19.72

Tooth types as in Table 1. Values for CA 770 and 771 have changed slightly since their original publication [30] due to an update in sectioning protocols [63]. Comparative fossil data are from [53, 67].

### Enamel-dentine junction morphology

**Fig 4:**
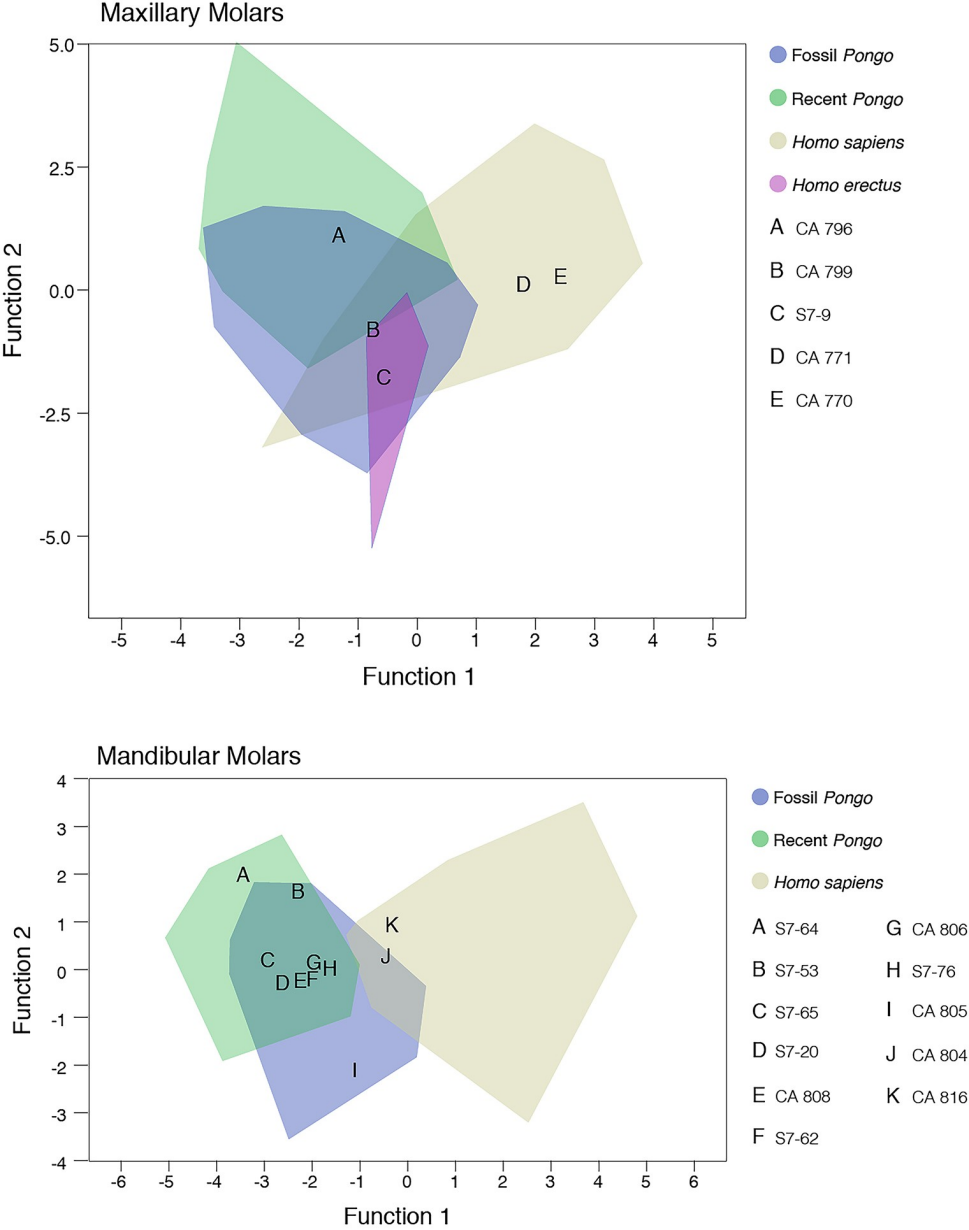
Plot depicting scores on the first two discriminant functions resulting from the DFA of maxillary (upper plot) and mandibular (lower plot) molar enamel-dentine junction shape metrics, and the position of the study sample. Note that there are no data available regarding enamel-dentine junction shape for mandibular molars of *H*. *erectus*.

Discriminant function analysis of molar EDJ landmark relative distances demonstrates that the *Homo* and *Pongo* comparative sample are grouped reliably at the generic level based on the nine distance ratios (91% of 247 maxillary teeth correctly classified; 96% of 247 mandibular teeth correctly classified). The main maxillary molar analysis had three significant functions with a combined *Χ*^2^ (24) = 387.4, Wilk’s λ = 0.199 (*p* < 0.001). After removal of the first function, there was still a strong association between groups and predictors: *Χ*^2^ (14) = 96.8, Wilk’s λ = 0.668 (*p* < 0.001). After removal of the second function, the significant relationship between groups and predictors persisted: *Χ*^2^ (6) = 19.7, Wilk’s λ = 0.921 (*p* = 0.003). A plot of the first two discriminant functions is shown in Fig 4. The first discriminant function accounts for 83.5% of the variance and has positive relationships with the buccal horn height relative to the center of the occlusal basin (landmark 5), the lingual horn height relative to the center of the occlusal basin, and the buccal dentine horn height. The second function accounts for 13.4% of the variance and has positive relationships with the width of the dentine midway between the crown base and the occlusal basin, the width of the dentine below the occlusal basin, the width of the dentine horns, and the lingual dentine horn height.

The discriminant function analysis of mandibular molars revealed two significant functions with a combined *Χ*^2^ (16) = 449.9, Wilk’s λ = 0.154 (*p* < 0.001). After removal of the first function, there was still a strong association between groups and predictors: *Χ*^2^ (7) = 65.2, Wilk’s λ = 0.763 (*p* < 0.001). The first discriminant function accounts for 92.7% of the variance and has positive relationships with the lingual dentine horn height, the buccal dentine horn height, the buccal horn height relative to the center of the occlusal basin, and the lingual horn height relative to the center of the occlusal basin. The second function accounts for 7.3% of the variance and has a positive relationship with the width of the dentine midway between the crown base and the occlusal basin, the width of the dentine below the occlusal basin, and the width of the dentine horns.

**Fig 5:**
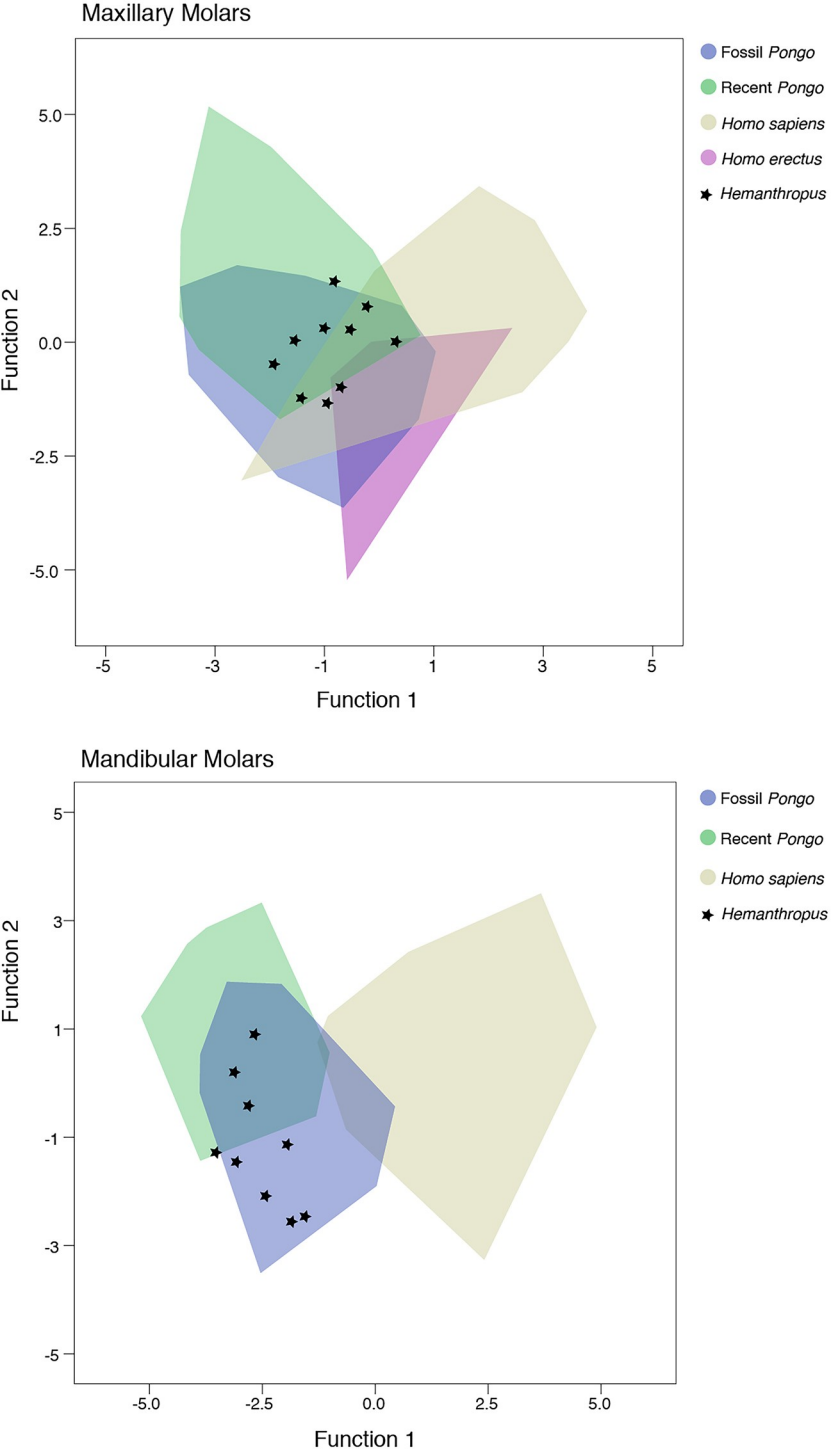
Plot depicting scores on the first two discriminant functions resulting from the DFA of maxillary (upper plot) and mandibular (lower plot) molar enamel-dentine junction shape metrics and position of 19 "*Hemanthropus peii*" molars.

Only three of 16 putative hominin or pongine molars from the Chinese Apothecary and Sangiran dome collections were classified as *Homo* when left as ungrouped cases (CA 770, CA 771, S7-9), falling within the range of *Homo sapiens* molars and outside the *Pongo* range in two of three instances (Fig 4). Two mandibular molars fell within the range of *H*. *sapiens* but were classified as *Pongo* as their most likely group (CA 804, CA 816). A similar DFA analysis of the "*Hemanthropus peii*" sample resulted in the classification of 14 molars as fossil *Pongo*, four molars as extant *Pongo* (including the type specimen, CA 673), and one maxillary molar (CA 705) as *H*. *sapiens* (Fig 5).

### Dental development

Long-period line periodicities in the Chinese Apothecary and Sangiran dome teeth range from 7 to at least 12 days (possibly 13 days) (Table 3). The periodicity of the *H*. *erectus* comparative sample ranges from 6–8 days (n = 7), which is significantly lower than the fossil *Pongo* sample (range 9–12 days, n = 19) (*Z* = -3.934, *p* < 0.001; calculated using the highest possible values for those *H*. *erectus* teeth that are uncertain). The *Sinanthropus officinalis* type (CA 770) has a periodicity of 8 days, which is within the range of the *H*. *erectus* sample, and below the fossil *Pongo* range. The value of the "*Hemanthropus peii*" type (CA 673) was somewhat indistinct, falling between 9–11 days, which exceeds the known range of *H*. *erectus*, but is within the fossil *Pongo* range. It was not possible to estimate the long-period line periodicity in two teeth from the study sample due to poor incremental feature visibility (S7-53, S7-76).

**Table 3:** Long-period line periodicity (in days).

Collection	Accession	Periodicity	Source
*Ungrouped Cases*			
CA	673	~9-11	This study
	770	8	" "
	771	7	" "
	772	7-8	" "
	796	9-11	" "
	804	~9-10	" "
	805	~12-13	" "
	806	8	" "
	807	11	" "
	808	10	" "
	816	9	" "
Sangiran	S7-9	8-9	" "
	S7-20	8	" "
	S7-62	9	" "
	S7-64	9-10	" "
	S7-65	7-8	" "
*Comparative samples*			
Zhoukoudian	M3549	8	" "
	M3550	7	" "
	M3887	8	" "
	PMU 25719	~7-8	" "
Trinil	11620	~6-7	30
	11621	6	30
Sangiran	S7-37	7	71
Fossil *Pongo*	n = 19	9.9 (9-12)	54; this study

**Fig 6:**
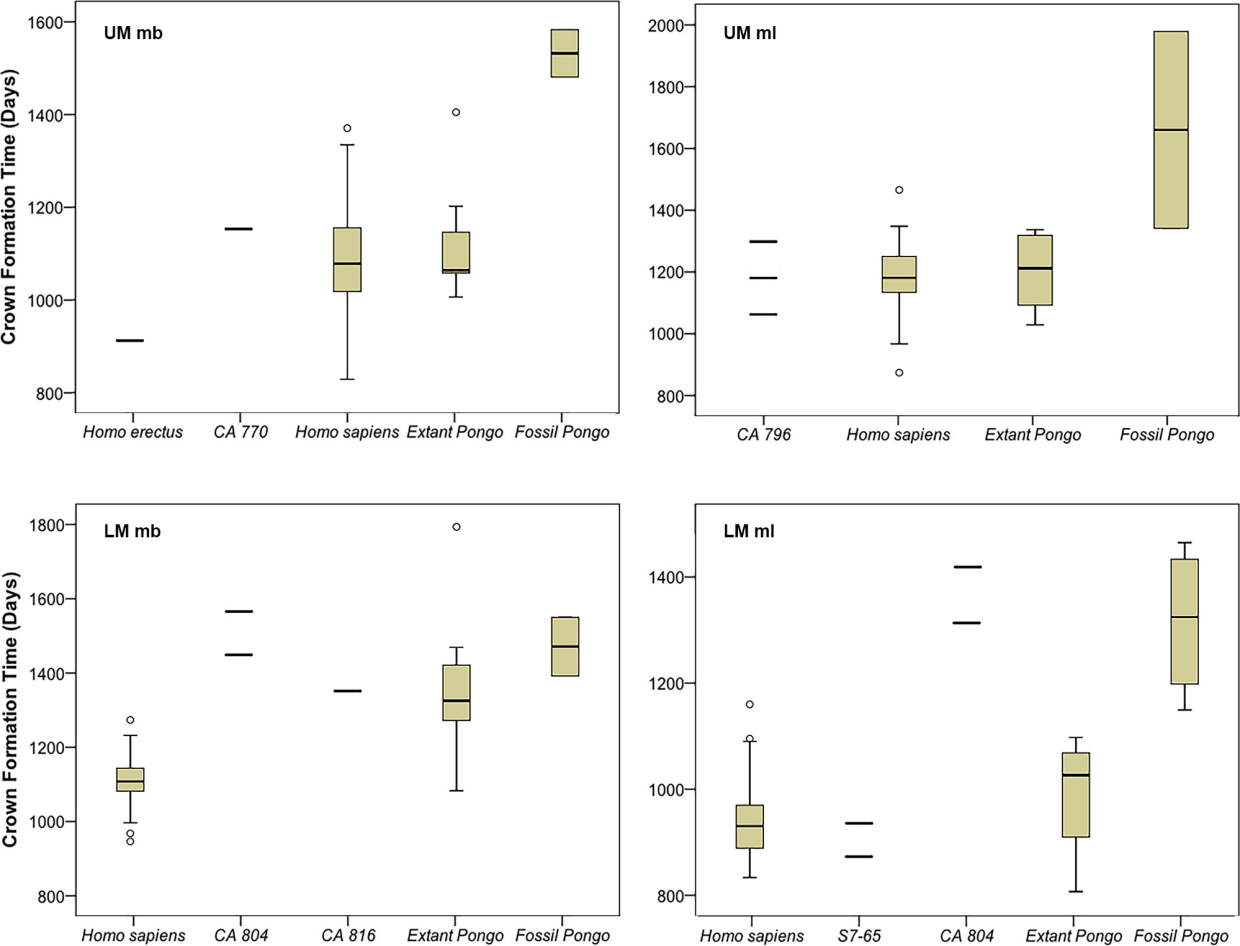
Crown formation times (days) in molar mesial cusps. UM = upper molar, LM = lower molar, mb = mesiobuccal cusp, ml = mesiolingual cusp. Note that first, second, and third molars have been combined for each cusp position due to uncertainty of the serial position of some fossil molars. Multiple estimates are presented for fossil samples with uncertain periodicity values (CA 796, CA 804, S7-65). *H*. *erectus* data (S7-37): [71]; *H*. *sapiens*: [72]; extant and fossil *Pongo*: [54] and this study.

Crown formation times were estimated for nine molar cusps of seven teeth in the Chinese Apothecary and Sangiran dome sample (Table 4), although three of these are minimum values (indicated by “>”) due to natural tooth wear. Four crown formation times are given as multiple values due to uncertainty in the long-period line periodicity (CA 796 mesiolingual cusp, CA 804 mesiobuccal and mesiolingual cusps, S7-65 mesiolingual cusp). Comparative samples are limited to one *H*. *erectus* upper first mesiobuccal cusp, which formed in approximately 2.4 years [71], and ten fossil *Pongo* molar cusps, which range from 3.2–5.4 years ([54] and this study]. Crown formation times for CA 770, CA 796, and S7-65 are similar to values for *Homo* (and extant *Pongo*), while CA 804 and CA 816 are similar to fossil *Pongo* (Fig 6).

**Table 4:** Crown formation times in the study sample.

Accession	Tooth	Cusp	Thick	C. Time	LP Number	Approach	Per	Lateral	CFT	CFT
			(microns)	(days)			(days)	(days)	(days)	(years)
CA 770	RUM1	mb	1315	313	105	cast	8	840	1153	3.16
CA 796	RUM1-2	ml	n/a	n/a	118 (dentine)	2D section	9-11	n/a	1062/1180/1298	2.91/3.23/3.56
CA 804	LLM1-2	mb	1659	395	117	2D section	9-10	n/a	1448/1565	3.97/4.29
" "	" "	" "	1475	351	107	" "	9-10		1314/1421	3.60/3.89
CA 806	LLM1-2	ml	>885	n/a	>82	2D section	8		>1100	>3.01
CA 816	RLM3	mb	950	226	125	3D model	9	1125	1351	3.70
S7-62	RLM1-2	mb	>1002	n/a	>107	2D section	9	>963	>1215	>3.32
" "	" "	ml	side	n/a	>67	2D + cast			>948	>2.60
S7-65	RLM2	ml	>1402	~432	~63	2D section	~7-8	~441/504	873/936	2.39/2.56

Tooth: types as in Table 1; Cusp: mb—mesiobuccal, ml—mesiolingual; Thick: cuspal thickness; C. Time: cuspal enamel formation time; LP Number: long-period line number, counted from casts, 2D sections, or 3D models (as indicated in Approach); Per: long-period line periodicity; Lateral: lateral formation time; CFT: cusp-specific crown formation time.

## Discussion

### Enamel structure

Enamel thickness appears to be of limited value for sorting hominin from pongine molars in mixed Asian faunas, as *Pongo* and *Homo* show overlapping ranges (see expanded extant data in [55, 57, 67]). This has been recognized since Miller's [27] radiographic study of the "Piltdown Man" jaw, which was ultimately determined to be a filed-down orangutan mandible [73]. Further complicating the issue is the presence of the Asian Pleistocene ape *Gigantopithecus blacki*; its relative enamel thickness values overlap thick-enameled fossil *Pongo* molars and most *Homo* specimens [36, 74] although its molar size and morphology are distinctive [14, 44]. Studies of the three-dimensional distribution of enamel throughout the crown may provide better taxonomic resolution, as *Pongo* molar enamel appears to be more uniformly distributed than enamel in *Homo* molars [36, 63, 64]. This approach will be less useful for worn teeth or those that show poor tissue contrast with microCT scanning, as was the case for much of the fossil sample studied here.

While the presence of moderately thick enamel (*sensu* Martin [28]) does not distinguish *Pongo* and *Homo* molars, enamel thickness at the low end of the *Pongo* range—such as majority of the "*Hemanthropus peii*" sample—is more consistent with non-hominin hominoid primates. This enigmatic fossil group appears to have relatively thinner enamel than Neanderthals, the thinnest-enameled members of the genus *Homo* [65, 67]. Evidence from molar enamel-dentine junction (EDJ) shape further supports their pongine status, as discriminant function analyses aligned 18 of 19 "*Hemanthropus peii*" molars with *Pongo* rather than *Homo*.

Our EDJ shape analysis confirms Martin's [28] observation that *Pongo* molars have shorter dentine horns than *Homo*, creating a flatter enamel-dentine junction and occlusal surface with less relief in the former taxon. Further study may clarify if there are other useful taxon-specific aspects of molar EDJ shape for sorting teeth in this region, as has been suggested by 3D analysis of maxillary molars of extant and fossil *Pongo* [75]. The small *H*. *erectus* maxillary molar comparative sample largely overlaps with both *Pongo* and recent *H*. *sapiens*, similar to the results of conventional metric and morphological analyses (reviewed in [30]). One tooth from our study sample showed greater affinities to *H*. *erectus* than to other groups (S7-9), and two were most like *H*. *sapiens* (CA 770, CA 771). These findings are consistent with information from hominin molar enamel thickness values, long-period line periodicities, and crown formation time (in the case of CA 770) (Table 5), which we discuss further below.

**Table 5:** Summary of structural and developmental information for the "*Hemanthropus peii*" type (CA 673) and Chinese Apothecary and Sangiran dome collections.

Accession	E Thick	EDJ	Periodicity	CFT	Affinity	Original Attribution
CA 673	Thin	*Pongo*	High	n/a	*Pongo*	*Hemanthropus peii* (type)
CA 770	Thick	*Homo*	Low	Intermediate	*Homo*	*Sinanthropus officinalis* (type)
CA 771	Intermediate	*Homo*	Low	n/a	*Homo*	*Sinanthropus officinalis*
CA 772	n/a	n/a	Low	n/a	*Homo*	*Sinanthropus officinalis*
CA 796	Thin	*Pongo*	High	Intermediate	*Pongo*	*Sinanthropus officinalis*
CA 799	Thin	*Pongo*	n/a	n/a	*Pongo*	*Sinanthropus officinalis*
CA 804	Thick	*Pongo*	Intermediate	High	*Pongo*	*Sinanthropus officinalis*
CA 805	Thin	*Pongo*	High	n/a	*Pongo*	*Sinanthropus officinalis*
CA 806	Intermediate	*Pongo*	Low	n/a	*likely Homo*	*Sinanthropus officinalis*
CA 807	n/a	n/a	High	n/a	*Pongo*	*Sinanthropus officinalis*
CA 808	Thick	*Pongo*	High	n/a	*Pongo*	*Sinanthropus officinalis*
CA 816	Intermediate	*Pongo*	Intermediate	High	*Pongo*	*Sinanthropus officinalis*
S7-9	Intermediate	*Homo*	Intermediate	n/a	*Homo*	*Homo erectus*
S7-20	Thick	*Pongo*	Low	n/a	*likely Homo*	probably fossil *Pongo*
S7-53	Thick	*Pongo*	n/a	n/a	*likely Pongo*	*Homo erectus*
S7-62	Intermediate	*Pongo*	Intermediate	n/a	*likely Pongo*	probably fossil *Pongo*
S7-64	Intermediate	*Pongo*	Intermediate	n/a	*likely Pongo*	*Homo erectus*
S7-65	Thick	*Pongo*	Low	Low	*Homo*	probably fossil *Pongo*
S7-76	Intermediate	*Pongo*	n/a	n/a	*likely Pongo*	*Homo erectus*

E thick: generalization of enamel thickness conditions (thin, intermediate, thick); EDJ: predictive results of discriminant function analysis; Periodicity: generalization of long-period line periodicity condition; (low, intermediate, high); CFT: generalization of crown formation time condition (low, intermediate, high); Affinity: dichotomous similarity of each tooth based on diagnostic conditions (highlighed in gray); Original Attribution of Chinese Apothecary (CA) teeth from von Koenigswald (recorded in the Senckenberg Research Institute and Natural History Museum Frankfurt Catalogue), and Sangiran (S7) teeth from Grine [5].

### Dental development

Developmental characters have been employed in taxonomic studies of hominins and hominoids for decades, but these studies have been complicated by methodological differences and a limited understanding of biological variation within and among serial tooth positions [39, 56]. Daily secretion rates, which vary among certain hominins [40], are similar in fossil *Pongo*, *H*. *erectus*, and recent *H*. *sapiens* (cuspal average = 4.11 μm/day: [56]). Long-period line periodicity may be more distinct, as fossil *Pongo* shows the highest average periodicity value of any primate, although a single *G*. *blacki* specimen has a reported periodicity of 11 days [74]. The average periodicity in our *H*. *erectus* comparative sample is similar to early *Homo* specimens from South Africa and also Neanderthals, which fall between 7–8 days [69, 72]. While expanded samples may lead to overlapping ranges for these fossil groups, periodicity values appear to be useful for distinguishing certain isolated *Pongo* and *Homo* teeth.

Crown formation times also vary between hominins and other hominoids (reviewed in [54, 69]). Fossil orangutans show the longest molar crown formation times, along with *G*. *blacki*, due in part to their elevated long-period line periodicities and large crown size [54, 74]. Limited data are available on early members of the genus *Homo*; post-canine formation times typically fall in the lower end of modern human ranges (Table 6). The sole exception to this is CA 770, which has a mesiobuccal cusp formation time that exceeds the known range of upper first molar values in modern humans.

**Table 6:** Post-canine crown formation times in fossil and extant *Homo*.

Tooth (cusp)	Early *Homo* (SA)	*Homo erectus*	CA 770	S7-65	*H*. *sapiens* (range)
UP4 (b)	n/a	986			966-1359
UM1 (mb)	n/a	912	1153		829-1074
UM1 (ml)	1035	n/a			967-1331
LP3 (b)	n/a	1173			1135-1614
LM1 (ml)	876	n/a			841-990
LM2 (ml)	n/a	n/a		873/936	834-1053

Tooth: types as in Table 1; Cusp: b—buccal; mb—mesiobuccal, ml—mesiolingual; Early *Homo* data from South Africa (SA): [69]; *H*. *erectus*: [71] and this study (M3549 LP3); recent *H*. *sapiens*: [69, 72].

### Presence and distribution of Asian hominins

Considerable efforts over the past 125 years have been dedicated to identifying hominins in Asian fossil assemblages, and this continues to the present day [16, 18, 30, 33, 41, 76–82]. It is now well established that *H*. *erectus* reached both mainland Asia and island Southeast Asia during the Early Pleistocene [83, 84]. The earliest recovered teeth of *H*. *erectus* (originally termed *Pithecanthropus erectus* and *Sinanthropus pekinensis*) from Trinil, Java and Zhoukoudian, China have been thoroughly discussed and debated (e.g., [85–87]). However, we concur with Ciochon [18] that the frequency of occurrence of *H*. *erectus* in Asia may have been overestimated due to misidentification of isolated pongine dental remains.

We have shown here and in [30] that consideration of internal structural and developmental characters reveal subtle differences between *H*. *erectus* and fossil *Pongo*, providing additional evidence that may be used in concert with examinations of molar crown size and external shape (also see [33, 41]). This approach could aid in the reassessment of dental remains from Tham Khuyen Cave, Vietnam [18, 76, 88] and Longgupo, China [4, 12, 89, 90], which were originally thought to belong to early *Homo* but have been recently reclassified as pongines by Ciochon [18]. Similarly, we suggest that von Koenigswald's Chinese Apothecary collection includes more pongine remains than has been previously appreciated, as information on long-period line periodicities and crown formation times is broadly consistent with results from EDJ shape and enamel thickness analyses.

This is particularly apparent when the "*Hemanthropus peii*" sample is compared with fossil *Pongo*. Wolpoff ([9]: p. 507) described this sample as "worn versions of the thick-enameled dental remains that have been attributed to "giant orangs."" Early Pleistocene fossil orangutans from China are especially large in comparison to modern-day orangutans, while Late Pleistocene forms are smaller [25]. Enamel thickness values and EDJ shapes that are nearly identical to *Pongo* call into question the proposition that the small "*Hemanthropus peii*" molar teeth belong to new Pleistocene hominoid (*contra* Ciochon [12, 18]). Moreover, the "*Hemanthropus peii*" type specimen shows a long-period periodicity value that is consistent with our fossil *Pongo* sample. While it is possible that additional pongine genera may be included in the Chinese Apothecary collection, or in other sites from mainland Asia [91], we concur with Harrison et al. [25] that the "*Hemanthropus peii*" teeth are most likely fossil orangutans.

The identification of pongine material from Sangiran is somewhat less secure. Grine and Franzen [5] were unable to agree on the presence of fossil orangutans in the surface finds made by G.H.R. von Koenigswald. Our analysis suggests that two of the teeth originally attributed to *Pongo* by Grine are more likely to be hominins (S7-20; S7-65), while three of the teeth originally attributed to *H*. *erectus* (S7-53; S7-64; S7-76) are more similar to orangutans, particularly in terms of their EDJ shape. This conclusion could be strengthened with an expansion of our *H*. *erectus* molar comparative sample, as the current one is limited to maxillary molars. Mandibular molar EDJ shapes of extant humans differ from those of extant and fossil orangutans, but it is possible that *H*. *erectus* mandibular molar EDJ shapes were less distinctive than those of extant humans. Faunal assemblages from this region have been suggested to indicate an open woodland environment that predates the arrival of orangutans on Java [92, 93]. Yet Drawhorn [94] notes that teeth in the Sangiran surface collection are unlikely to derive from a single biological population, reaching the same conclusion as Grine that some of the teeth derive from fossil orangutans. A recent analysis of enamel thickness and EDJ shape in a deciduous molar from Sangiran concluded that it belongs to *Pongo* [41], lending further support to the idea that von Koenigswald’s Javanese collection contains both hominin and pongine material.

While structural and developmental integration may be useful for generic distinction of primate teeth, it remains to be seen whether these characters will aide in the distinction of Asian hominins, as is the case for comparisons of modern humans and Neanderthals (e.g., [65, 67, 72]). Smith et al. [67] documented considerable variation in enamel thickness within members of the genus *Homo*, with Neanderthals being an especially distinct group. Similarly, Neanderthals show lower long-period line periodicities and faster extension rates than recent humans, leading to shorter crown formation times [72]. Developmental analyses of Asian hominin teeth are limited to studies of the *H*. *erectus* Sangiran maxillary fragment S7-37 [71] and the Trinil molars [30], as well as an enigmatic child's maxilla from Xujiayao, China [66, 79, 95]. Fossils from this latter individual are described as possessing a combination of structural traits that are more similar to early Pleistocene *Homo* and Neanderthal fossils than to modern humans, yet the distribution and number of long-period growth lines are most similar to recent humans.

It is possible that at least two and as many as four *Homo* species overlapped in Southeast Asia during the Pleistocene. Multiple lines of evidence place *H*. *sapiens* in China before 70,000 years ago [96, 97], which may be concurrent with the enigmatic hominin material from Xujiayao [66]. *Homo sapiens* is now known to have crossed island Southeast Asia 63–73,000 years ago, reaching Australia by at least 65,000 years ago [81, 98]. Moreover, a hominin toe bone from the Philippines at 67,000 years ago has been provisionally attributed to a small-bodied *H*. *sapiens* individual [99]. These groups may have encountered other hominins such as *Homo floresiensis*, which survived on Flores until 50,000–60,000 years ago [100]. Even if *H*. *erectus*, *H*. *floresiensis*, *H*. *sapiens* or other hominins did not occupy the same areas at the same time, uncertainties in dating, geological and taphonomic processes, and the provenience of mixed material such as von Koenigswald's Chinese Apothecary collection underscore the need to reassess the identification of other isolated, large-bodied, primate dental remains from the Pleistocene of Asia.

## Supporting information

S1 File Contains supporting tables, figures, and references. Table A. Asian Pleistocene sites with isolated dental remains reported to belong to Hominins. Fig A. Chinese Apothecary dental material attributed to "*Hemanthropus peii*" by von Koenigswald. Fig B. Chinese Apothecary dental material attributed to *Sinanthropus officinalis* (= *Homo erectus*) by von Koenigswald. Fig C. Sangiran dental material attributed to *Homo erectus* or fossil *Pongo* by Grine included in this study.
(DOC)

## References

[1] Oakley KP, Campbell BG, Molleson TI, editors. Catalogue of fossil hominids part III: Americas, Asia, Australasia London: British Museum (Natural History); 1975.

[2] Wu X, Poirier FE. Human evolution in China New York: Oxford University Press; 1995.

[3] Antón S. Natural history of *Homo erectus*. Yearb Phys Anthropol. 2003; 46:126–170. 10.1002/ajpa.10399 14666536

[4] Huang W, Ciochon RL, Gu Y, Larick R, Fang Q, Yonge C, et al Early *Homo* and associated artefacts from Asia. Nature 1995; 378: 275–278. 10.1038/378275a0 7477345

[5] Grine F, Franzen JL. Fossil hominid teeth from the Sangiran Dome (Java, Indonesia). Courier Forschungsinstitut Senckenberg 1994; 171: 75–103.

[6] Ciochon RL, Long VT, Larick R, Gonzalez L, R, Vos J de, et al Dated co‑occurrence of *Homo erectus* and *Gigantopithecus* from Tham Khuyen Cave, Vietnam. Proc Natl Acad Sci U S A 1996; 93: 3016–3020. 8610161 10.1073/pnas.93.7.3016 PMC39753

[7] Hooijer DA. Prehistoric teeth of man and of the orangutan from central Sumatra, with notes on the fossil orangutan from Java and Southern China. Zool Meded. 1948; 29: 175–301.

[8] Koenigswald GHR von. De *Pithecanthropus*‑kiezen uit de collectie DUBOIS. Proc Roy Acad Amsterdam 1967; 76: 42–45.

[9] Wolpoff MH. *Ramapithecus* and hominid origins. Curr Anthropol. 1982; 23: 501–522.

[10] Wanpo H, Ciochon R, Yumin G, Larick R, Qiren F, Schwarcz H, et al Early *Homo* and associated artefacts from Asia. Nature 1995; 378: 275–278. 10.1038/378275a0 7477345

[11] Schwartz JH, Tattersall I. The human fossil record, volume two New York: Wiley‑Liss; 2003.

[12] Ciochon R. The mystery ape of Pleistocene Asia. Nature 2009; 459: 910–911. 10.1038/459910a 19536242

[13] Koenigswald GHR von. Eine fossile Säugetierfauna mit Simia aus Südchina. Proc Roy Acad Amsterdam 1935; 38: 872–879.

[14] Koenigswald GHR von. Remarks on *Gigantopithecus* and other hominid remains from South China. Proc Roy Acad Amsterdam Ser B, 1957; 60: 153–59.

[15] Koenigswald GHR von. Die fossilen Säugetierfauna Javas. Proc Roy Acad Amsterdam 1935; 38: 188–198.

[16] Wang W, Potts R, Hou Y, Chen Y, Wu H, Yuan B, et al Early Pleistocene hominid teeth recovered in Mohui cave in Bubing Basin, Guangxi, South China. Chin Sci Bull. 2005; 50: 2777–2782.

[17] Wang W, Potts R, Baoyin Y, Huang W, Cheng H, Edwards RL, et al Sequence of mammalian fossils, including hominoid teeth, from the Bubing Basin caves, South China. J Hum Evol. 2007; 52: 370–379. 10.1016/j.jhevol.2006.10.003 17198721

[18] Ciochon R. Divorcing hominins from the Stegodon-Ailuropoda fauna: new views on the antiquity of hominins in Asia In: Fleagle JG et al, editors. Out of Africa I: The first hominin colonization of Eurasia. Dordrecht: Springer; 2010 pp. 111–126.

[19] Koenigswald GHR von. Erratum: *Hemanthropus* n. g. not *Hemianthropus*. Proc Roy Acad Amsterdam Ser B 1957; 60: 416.

[20] Simons EL. Some fallacies in the study of hominid phylogeny. Science 1963; 141: 879–889. 14043325 10.1126/science.141.3584.879

[21] Koenigswald GHR von. *Australopithecus*, *Meganthropus*, and *Ramapithecus*. J Hum Evol. 1973; 2: 487–491.

[22] Tobias PV, Koenigswald GHR von. A comparison between the Olduvai hominines and those of Java and some implications for hominid phylogeny. Nature 1965; 204: 515–518. 10.1038/204515a0 14238152

[23] Pope GG. Evidence on the age of the Asian Hominidae. Proc Natl Acad Sci U S A 1983; 80: 4988–4992. 6410399 10.1073/pnas.80.16.4988 PMC384173

[24] McKenna MC, Bell SK. Classification of mammals: above the species level New York: Columbia University Press; 1997.

[25] Harrison T, Jin C, Zhang Y, Wang Y, Zhu M. Fossil *Pongo* from the Early Pleistocene *Gigantopithecus* fauna of Chongzuo, Guangxi, southern China. Quat Int. 2014; 354: 59–67.

[26] Pilloud MA, Maier C, Scott GR, Edgar HJH. Molar crenulation trait definition and variation in modern human populations. HOMO—Journal of Comparative Human Biology 2018; 69: 77–85. 10.1016/j.jchb.2018.06.001 30007496

[27] Miller GS. The Piltdown jaw. Am J Phys Anthropol. 1918; 1: 25–52.

[28] Martin LB. Relationships of the later Miocene Hominoidea. Ph.D. Dissertation, The University College London. 1983.

[29] Beynon AD, Wood BA. Variations in enamel thickness and structure in east African hominids. Am J Phys Anthropol. 1986; 70: 177–193. 10.1002/ajpa.1330700205 3090891

[30] Smith TM, Olejniczak AJ, Kupczik K, Lazzari V, Vos J de, Kullmer O et al (2009) Taxonomic assessment of the Trinil molars using non-destructive 3D structural and development analysis. PaleoAnthropol. 2009: 117–129.

[31] Benazzi S, Douka K, Fornai C, Bauer CC, Kullmer O, Svoboda J, et al Early dispersal of modern humans in Europe and implications for Neanderthal behaviour. Nature 2011; 479: 525–528. 10.1038/nature10617 22048311

[32] Benazzi S, Viola B, Kullmer O, Fiorenza L, Harvati K, Paul T, et al A reassessment of the Neanderthal teeth from Taddeo cave (southern Italy). J Hum Evol. 2011; 61: 377–387. 10.1016/j.jhevol.2011.05.001 21683429

[33] Zanolli C, Bondioli L, Mancini L, Mazurier A, Widianto H, Macchiarelli R. Brief communication: two human fossil deciduous molars from the Sangiran Dome (Java, Indonesia): outer and inner morphology. Am J Phys Anthropol. 2012; 147: 472–481. 10.1002/ajpa.21657 22281866

[34] Olejniczak AJ, Martin LB, Ulhaas L. Quantification of dentine shape in anthropoid primates. Ann Anat. 2004; 186: 479–485. 10.1016/S0940-9602(04)80087-6 15646281

[35] Olejniczak AJ, Gilbert CC, Martin LB, Smith TM, Ulhaas L, Grine FE. Morphology of the enamel-dentine junction in sections of anthropoid primate maxillary molars. J Hum Evol. 2007; 53: 292–301. 10.1016/j.jhevol.2007.04.006 17582465

[36] Olejniczak AJ, Smith TM, Wang W, Potts R, Ciochon R, Kullmer O, et al Molar enamel thickness and dentine horn height in *Gigantopithecus blacki*. Am J Phys Anthropol. 2008; 135: 85–91. 10.1002/ajpa.20711 17941103

[37] Skinner MM, Wood BA, Boesch C, Olejniczak AJ, Rosas A, Smith TM et al Dental trait expression at the enamel‑dentine junction of lower molars in extant and fossil hominoids. J Hum Evol. 2008; 54: 173–186. 10.1016/j.jhevol.2007.09.012 18048081

[38] Skinner MM, Gunz P, Wood BA, Boesch C, Hublin J-J. Discrimination of extant *Pan* species and subspecies using the enamel–dentine junction morphology of lower molars. Am J Phys Anthropol. 2009; 140: 234–243. 10.1002/ajpa.21057 19382140

[39] Smith TM. Incremental dental development: methods and applications in hominoid evolutionary studies. J Hum Evol. 2008; 54: 205–224. 10.1016/j.jhevol.2007.09.020 18045649

[40] Lacruz RS, Dean MC, Ramirez-Rozzi F, Bromage TG. Megadontia, striae periodicity and patterns of enamel secretion in Plio-Pleistocene fossil hominins. J Anat. 2008; 213: 148–158. 10.1111/j.1469-7580.2008.00938.x 19172730 PMC2526111

[41] Zanolli C, Grine FE, Kullmer O, Schrenk F, Macchiarelli R. Brief communication: the early Pleistocene deciduous hominid molar FS-72 from the Sangiran dome of Java, Indonesia: a taxonomic reappraisal based on its comparative endostructural characterization. Am J Phys Anthropol. 2015; 157: 666–674. 10.1002/ajpa.22748 25845703

[42] Zanolli C, Pan L, Dumoncel J, Kullmer O, Kundrát M, Liu W, et al Inner tooth morphology of *Homo erectus* from Zhoukoudian. New evidence from an old collection housed at Uppsala University, Sweden. J Hum Evol. 2018; 116: 1–13. 10.1016/j.jhevol.2017.11.002 29477178

[43] Zanolli C. Brief communication: molar crown inner structural organization in Javanese *Homo erectus*. Am J Phys Anthropol. 2015; 156: 148–157. 10.1002/ajpa.22611 25209431

[44] Koenigswald GHR von. *Gigantopithecus blacki* VON KOENIGSWALD, a giant fossil hominoid from the Pleistocene of Southern China. Anthropol Pap Am Mus Nat Hist. 1952; 43: 310–325.

[45] Dubois E. Palaeontologische onderzoekingen op Java. Extra bijvoegsel der Javasche Courant, Verslag van het Mijnwezen over het 3e kwartaal 1891. 1892; 12–14.

[46] Dubois E. Pithecanthropus erectus, einen menschenaehnliche Uebergangsform aus Java Batavia: Landesdruckerei; 1894.

[47] Dubois E. Pithecanthropus erectus, einen menschenaehnliche Uebergangsform. In: Compte-rendu des séances du troisième Congrès International de Zoologie, Leyde, 16‑21 Septembre, 1895 Leiden: E.J. Brill; 1896 pp. 251–271, pl. 2.

[48] Black D. On a lower molar hominid tooth from the Chou Kou Tien deposit. Palaeontol Sinica Ser D. 1927; 7: 1–28.

[49] Koenigswald GHR, Weidenreich F. The relationship between *Pithecanthropus* and *Sinanthropus*. Nature 1939; 144: 926–929.

[50] Zdansky O. Preliminary notice on two teeth of a hominid from a cave in Chiihli (China). Bull Geol Soc China 1927; 5: 281–284.

[51] Zdansky O. A new tooth of *Sinanthropus pekinensis* Black. Acta Zool. 1952; 33: 189–191.

[52] Kundrát M, Liu W, Ebbestad JOR, Ahlberg P, Tong H. New tooth of Peking Man recognized in laboratory at Uppsala University. Acta Anthropol Sinica 2015; 34: 1–14.

[53] Smith TM, Bacon A-M, Demeter F, Kullmer O, Nguyen KT, Vos J de, et al 2011 Dental tissue proportions in fossil orangutans from mainland Asia and Indonesia. Hum Origins Res, 2011; 1: e1–e6.

[54] Smith TM. Dental development in living and fossil orangutans. J Hum Evol 2016; 94: 92–105. 10.1016/j.jhevol.2016.02.008 27178461

[55] Smith TM, Olejniczak AJ, Reid DJ, Ferrell RJ, Hublin J‑J. Modern human molar enamel thickness and enamel‑dentine junction shape. Arch Oral Biol. 2006; 51: 974–995. 10.1016/j.archoralbio.2006.04.012 16814245

[56] Smith TM, Reid DJ, Dean MC, Olejniczak AJ, Ferrell RJ, Martin LB. New perspectives on chimpanzee and human molar crown development In: Bailey S, Hublin J-J, editors. Dental perspectives on human evolution: state of the art research in dental paleoanthropology. Dordrecht: Springer; 2007 pp. 177–192.

[57] Smith TM, Kupczik K, Machanda Z, Skinner MM, Zermeno JP. 2012. Enamel thickness in Bornean and Sumatran orangutan dentitions. Am J Phys Anthropol. 2012; 147: 417–426. 10.1002/ajpa.22009 22271572

[58] Olejniczak AJ, Grine FE. Assessment of the accuracy of dental enamel thickness measurements using micro‑focal X‑ray computed tomography. Anat Rec. 2006; 288A: 263–275. 10.1002/ar.a.20307 16463379

[59] Smith TM, Tafforeau P. New visions of dental tissue research: tooth development, chemistry, and structure. Evol Anthropol. 2008; 17: 213–226.

[60] Tafforeau PT, Smith TM. Nondestructive imaging of hominoid dental microstructure using phase contrast X-ray synchrotron microtomography. J Hum Evol. 2008; 54: 272–278. 10.1016/j.jhevol.2007.09.018 18045654

[61] Immel A, Le Cabec A, Bonazzi M, Herbig A, Temming H, Schuenemann VJ, et al Effect of X-ray irradiation on ancient DNA in sub-fossil bones–Guidelines for safe X-ray imaging. Sci Rep. 2016; 6: 32969 10.1038/srep32969 27615365 PMC5018823

[62] Lyckegaard A, Johnson G, Tafforeau P. Correction of ring artifacts in X-ray tomographic images. Int J Tomo Stat 2011; 18: 1–9.

[63] Kono R. Molar enamel thickness and distribution patterns in extant great apes and humans: new insights based on a 3‑dimensional whole crown perspective. Anthropol Sci. 2004; 112: 121–146.

[64] Tafforeau P. Phylogenetic and functional aspects of tooth enamel microstructure and three‑dimensional structure of modern and fossil primate molars. Ph.D. Dissertation, Université de Montpellier II. 2004.

[65] Olejniczak AJ, Smith TM, Feeney RNM, Macchiarelli R, Mazurier A, Bondioli L, et al Dental tissue proportions and enamel thickness in Neandertal and modern human molars. J Hum Evol. 2008; 55: 12–23. 10.1016/j.jhevol.2007.11.004 18321561

[66] Xing S, Martinón-Torres M, Bermúdez de Castro JM, Wu X, Liu W. Hominin teeth from the early Late Pleistocene site of Xujiayao, northern China. Am J Phys Anthropol. 2015; 156:224–240. 10.1002/ajpa.22641 25329008

[67] Smith TM, Olejniczak AJ, Zermeno JP, Tafforeau P, Skinner MM, et al Variation in enamel thickness within the genus *Homo*. J Hum Evol. 2012b; 62: 395–411. 10.1016/j.jhevol.2011.12.004 22361504

[68] Martin LB. Significance of enamel thickness in hominoid evolution. Nature 1985; 314: 260–263. 3920525 10.1038/314260a0

[69] Smith TM, Tafforeau P, Le Cabec A, Bonnin A, Houssaye A, Pouech J, et al Dental ontogeny in Pliocene and early Pleistocene hominins. PLoS One 2015; 10: e0118118 10.1371/journal.pone.0118118 25692765 PMC4334485

[70] Le Cabec A, Tang N, Tafforeau P. Accessing developmental information of fossil hominin teeth using new synchrotron microtomography-based visualization techniques of dental surfaces and interfaces. PLoS One 2015; 10.1371/journal.pone.0115511 PMID: 25616135 PMC4406681 25901602

[71] Dean C, Leakey MG, Reid D, Schrenk F, Schwartz GT, Stringer C, et al Growth processes in teeth distinguish modern humans from *Homo erectus* and earlier hominins. Nature 2001; 414: 628–631. 10.1038/414628a 11740557

[72] Smith TM, Tafforeau P, Reid DJ, Pouech J, Lazzari V, Zermeno JP, et al Dental evidence for ontogenetic differences between modern humans and Neanderthals. Proc Natl Acad Sci U S A 2010; 107: 20923–20928. 10.1073/pnas.1010906107 21078988 PMC3000267

[73] De Groote I, Flink LG, Abbas R, Bello SM, Burgio L, Buck LT, et al New genetic and morphological evidence suggests a single hoaxer created ‘Piltdown Man.’ R. Soc. open sci. 2016 3: 160679 10.1098/rsos.160679 27853612 PMC5108962

[74] Dean MC, Schrenk F. Enamel thickness and development in a third permanent molar of *Gigantopithecus blacki*. J Hum Evol. 2003; 45: 381–387. 14624748 10.1016/j.jhevol.2003.08.009

[75] Ortiz A, Bailey SE, Delgado M, Zanolli C, Demeter F, Bacon A-M, et al *Homo* or *Pongo*? Trigon morphology of maxillary molars may solve taxonomic controversies over isolated hominoid teeth from the Asian Pleistocene. ESHE Abstract 2017; p. 140 http://eshe.eu/static/eshe/files/PESHE/PESHE_2017_FINAL.pdf

[76] Demeter F, Bacon AM, Nguyen KT, Long VT, Matsumura H, Ha HN, et al An archaic *Homo* molar from Northern Vietnam. Curr Anthropol. 2004; 45: 535–541.

[77] Demeter F, Bacon AM, Nguyen KT, Long VT, Duringer P, Roussé S et al Discovery of a second human molar and cranium fragment in the late Middle to Late Pleistocene cave of Ma U’Oi (Northern Vietnam). J Hum Evol. 2005; 48: 393–402. 10.1016/j.jhevol.2004.12.004 15788185

[78] Xing S, Martinón-Torres M, Bermúdez de Castro JM, Zhang Y, Fan X, Zheng L, et al Middle Pleistocene hominin teeth from Longtan Cave, Hexian, China. PLoS One 2014; 9: e114265 10.1371/journal.pone.0114265 25551383 PMC4281145

[79] Xing S, Guatelli-Steinberg D, O’Hara M, Wu X, Liu W, Reid DJ. Perikymata distribution in *Homo* with special reference to the Xujiayao juvenile. Am J Phys Anthropol. 2015; 157: 684–693. 10.1002/ajpa.22760 26059551

[80] Xing S, Sun C, Martinón-Torres M, Bermúdez de Castro JM, Han F, Zhang Y, et al Hominin teeth from the Middle Pleistocene site of Yiyuan, Eastern China. J Hum Evol. 2016; 95: 33–54. 10.1016/j.jhevol.2016.03.004 27260173

[81] Westaway KE, Louys J, Due Awe R, Morwood MJ, Price GJ, Zhao J -x. et al An early modern human presence in Sumatra 73,000–63,000 years ago. Nature 2017; 548: 322–325. 10.1038/nature23452 28792933

[82] Martinón-Torres M, Wu X, Bermúdez de Castro JM, Xing S, Liu W. *Homo sapiens* in the Eastern Asian Late Pleistocene. Curr Anthropol. 2017; 58: S434–S448.

[83] Swisher CC, Curtis GH, Jacob T, Getty AG, Suprijo A, Widiasmoro. Age of the earliest known hominids in Java, Indonesia. Science 1994; 263: 1118–1121. 8108729 10.1126/science.8108729

[84] Zhu Z, Dennell R, Huang W, Wu Y, Qiu S, Yang S, et al Hominin occupation of the Chinese Loess Plateau since about 2.1 million years ago. Nature 2018; 559: 608–612. 10.1038/s41586-018-0299-4 29995848

[85] Weidenreich F. The dentition of *Sinanthropus pekinensis*: a comparative odontography of the hominids. Palaeontologia Sinica. New Series D. 1937; No. 1. Whole Series 101: 1–180.

[86] Janssen Groesbeek B. The serial position of the Trinil upper molars. Anthropol Sci. 1996; 104: 107–130.

[87] Wang Q, Sun L. Eightieth year of Peking Man: Current status of Peking Man and the Zhoukoudian site. Anthropol Rev. 2000; 63: 19–30.

[88] Harrison T. Archaeological and ecological implications of the primate fauna from prehistoric sites in Borneo. Indo-Pacific Prehistory Association Bulletin (Melaka Papers, Volume 4) 2000; 20: 133–146.

[89] Etler DA. Mystery ape: other fossils suggest that it’s no mystery at all. Nature 2009; 460: 684. 10.1038/460684a 19661893

[90] Padian K. Mystery ape: a call for taxonomic rigour. Nature 2009; 460: 684. 10.1038/460684b 19661894

[91] Schwartz JH, Long VT, Cuong NL, Kha LT, Tattersall I. A review of the Pleistocene hominoid fauna of the socialist republic of Vietnam (excluding Hylobatidae). Anthropol Pap Am Mus Nat Hist 1995; 76: 1–24.

[92] Vos J de. Faunal stratigraphy and correlation of the Indonesian hominid sites In: Delson E. editor. Ancestors: the hard evidence. New York: Alan R. Liss; 1985 pp. 215–220.

[93] Bergh GD van den, Vos J de, Sondaar PY. The Late Quaternary palaeogeography of mammal evolution in the Indonesian Archipelago. Palaeogeogr Palaeoclimatol Palaeoecol. 2001; 171: 385–408.

[94] Drawhorn GM. The systematics and paleodemography of fossil orangutans (genus Pongo). Ph.D. Dissertation, The University of California, Davis. 1995.

[95] Xing S, Guatelli-Steinberg D, O’Hara M, Li J, Wei P, Liu W. Micro-CT imaging and analysis of enamel defects on the Early Late Pleistocene Xujiayao juvenile. Int J Osteoarchaeol. 2015; 10.1002/oa.2504

[96] Liu W, Martinón-Torres M, Cai J, Xing S, Tong H, Pei S, et al The earliest unequivocally modern humans in southern China. Nature 2015; 526: 696–699. 10.1038/nature15696 26466566

[97] Michel V, Valladas H, Shen G, Wang W, Zhao J, Shen C-C, et al The earliest modern *Homo sapiens* in China? J Hum Evol. 2016; 101: 101–104. 10.1016/j.jhevol.2016.07.008 27586079

[98] Clarkson C, Jacobs Z, Marwick B, Fullagar R, Wallis L, Smith M, et al Human occupation of northern Australia by 65,000 years ago. Nature 2017; 547: 306–310. 10.1038/nature22968 28726833

[99] Mijares AS, Détroit F, Piper P, Grü R, Bellwood P, Aubert M, et al New evidence for a 67,000-year-old human presence at Callao Cave, Luzon, Philippines. J Hum Evol. 2010; 59: 123–132. 10.1016/j.jhevol.2010.04.008 20569967

[100] Sutikna T, Tocheri MW, Morwood MJ, Saptomo EW, Jatmiko, Due Awe R, et al Revised stratigraphy and chronology for *Homo floresiensis* at Liang Bua in Indonesia. Nature 2016; 532: 366–369. 10.1038/nature17179 27027286

