# Supplementary material for: Disentangling isolated dental remains of Asian Pleistocene hominins and pongines
Source: PLoS One. 2018 Nov 1;13(11):e0204737. doi: 10.1371/journal.pone.0204737 (PMC6211657; doi:10.1371/journal.pone.0204737)
Supplement: S1 File — Table A. Asian Pleistocene sites with isolated dental remains reported to belong to Hominins. Fig A. Chinese Apothecary dental material attributed to "Hemanthropus peii" by von Koenigswald. Fig B. Chinese Apothecary dental material attributed to Sinanthropus officinalis (= Homo erectus) by von Koenigswald. Fig C. Sangiran dental material attributed to Homo erectus or fossil Pongo by Grine included in this study. (DOC) [file pone.0204737.s001.doc]

**Table A. Asian Pleistocene sites with isolated dental remains reported to belong to hominins.**

**Figure A. Chinese Apothecary dental material attributed to "*Hemanthropus peii*" by von Koenigswald.**


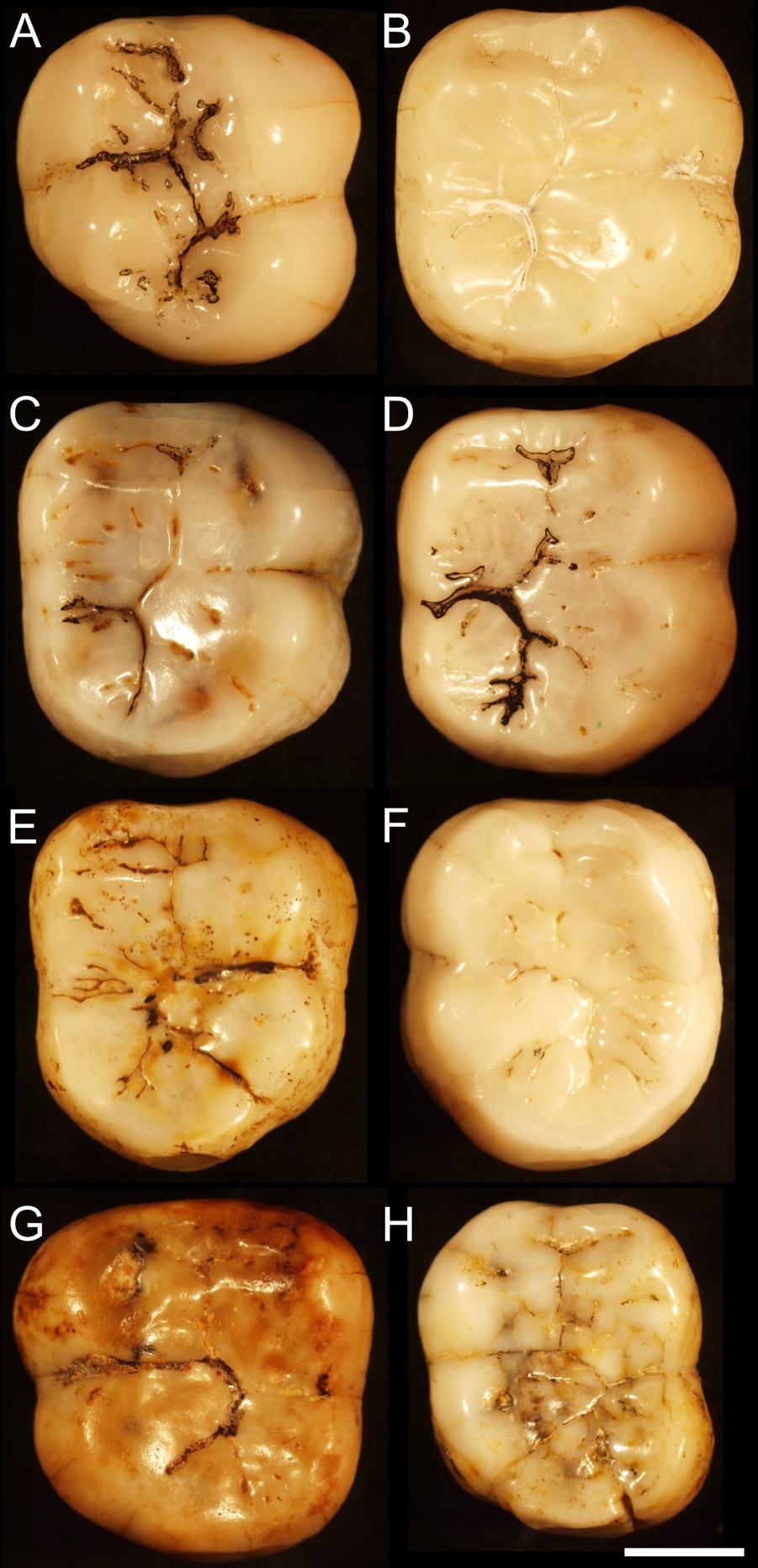


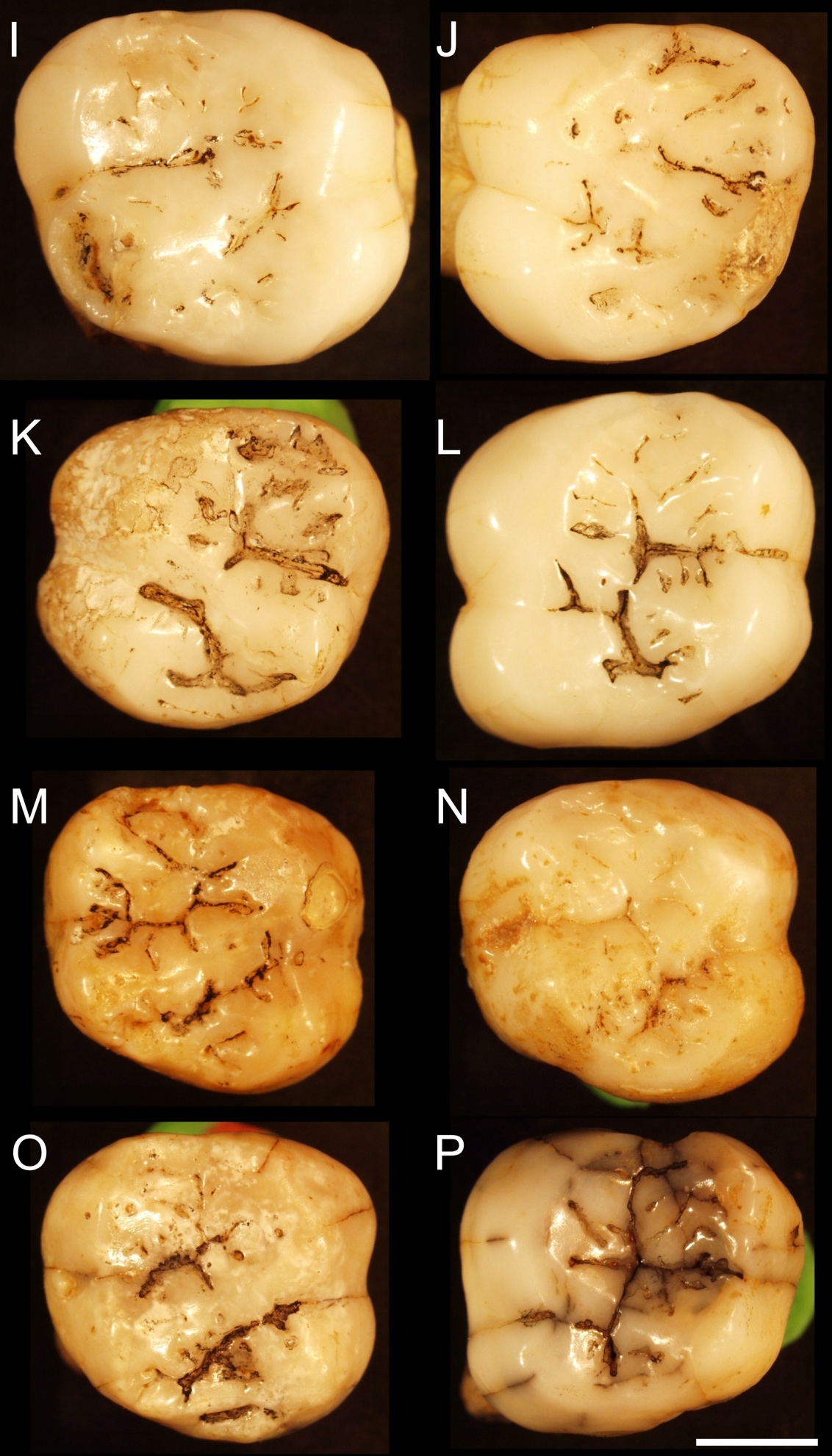


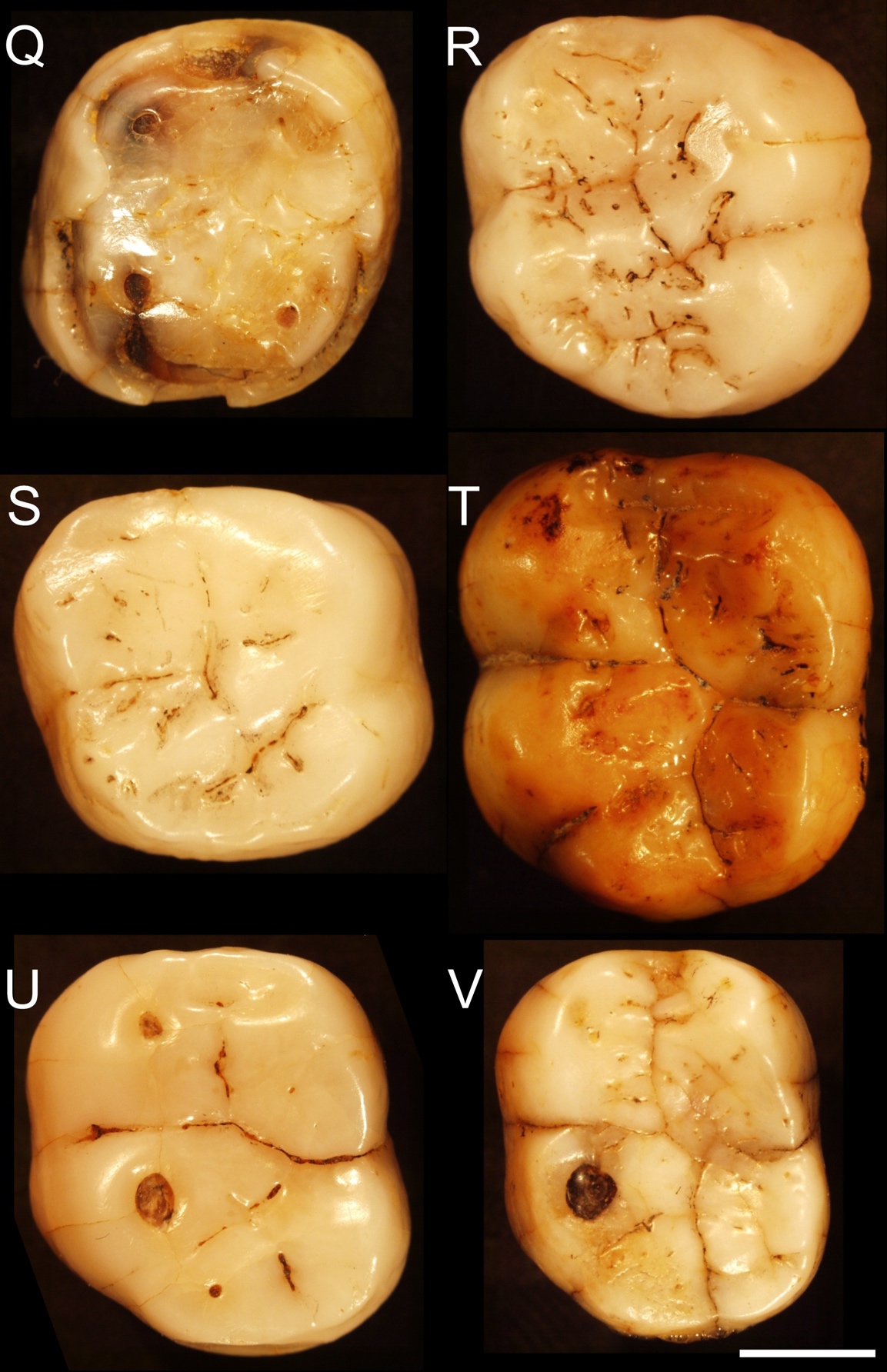


(A) 673 (type), (B) 674, (C) 675, (D) 677, (E) 678, (F) 685, (G) 686, (H) 689, (I) 690, (J) 692, (K) 695, (L) 696, (M) 703, (N) 704, (O) 705, (P) 707, (Q) 709, (R) 724, (S) 725, (T) 728, (U) 729, (V) 730. Scale bar is equal to 5 mm.

**Figure B. Chinese Apothecary dental material attributed to *Sinanthropus officinalis* (= *Homo erectus*) by von Koenigswald.**


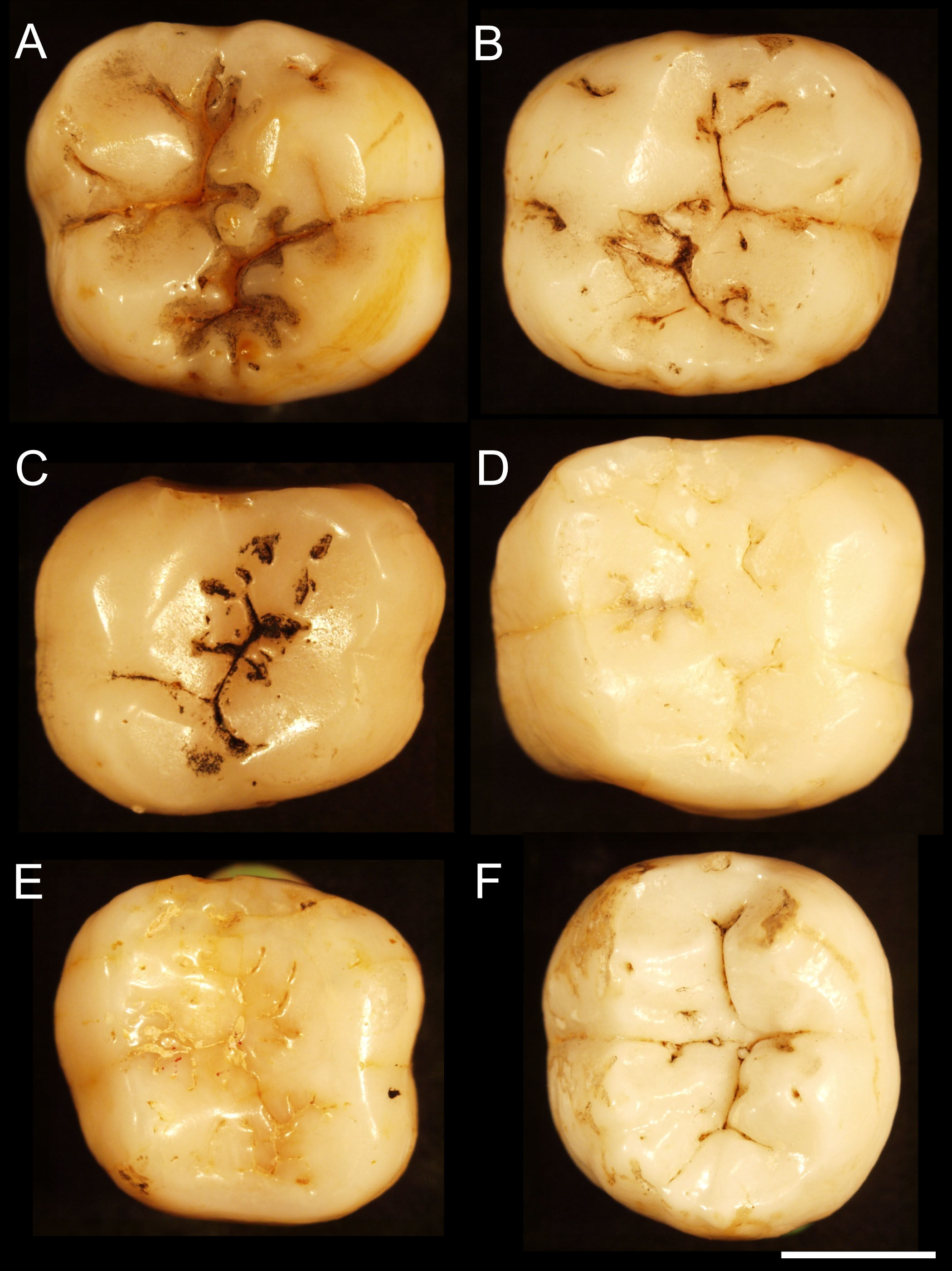


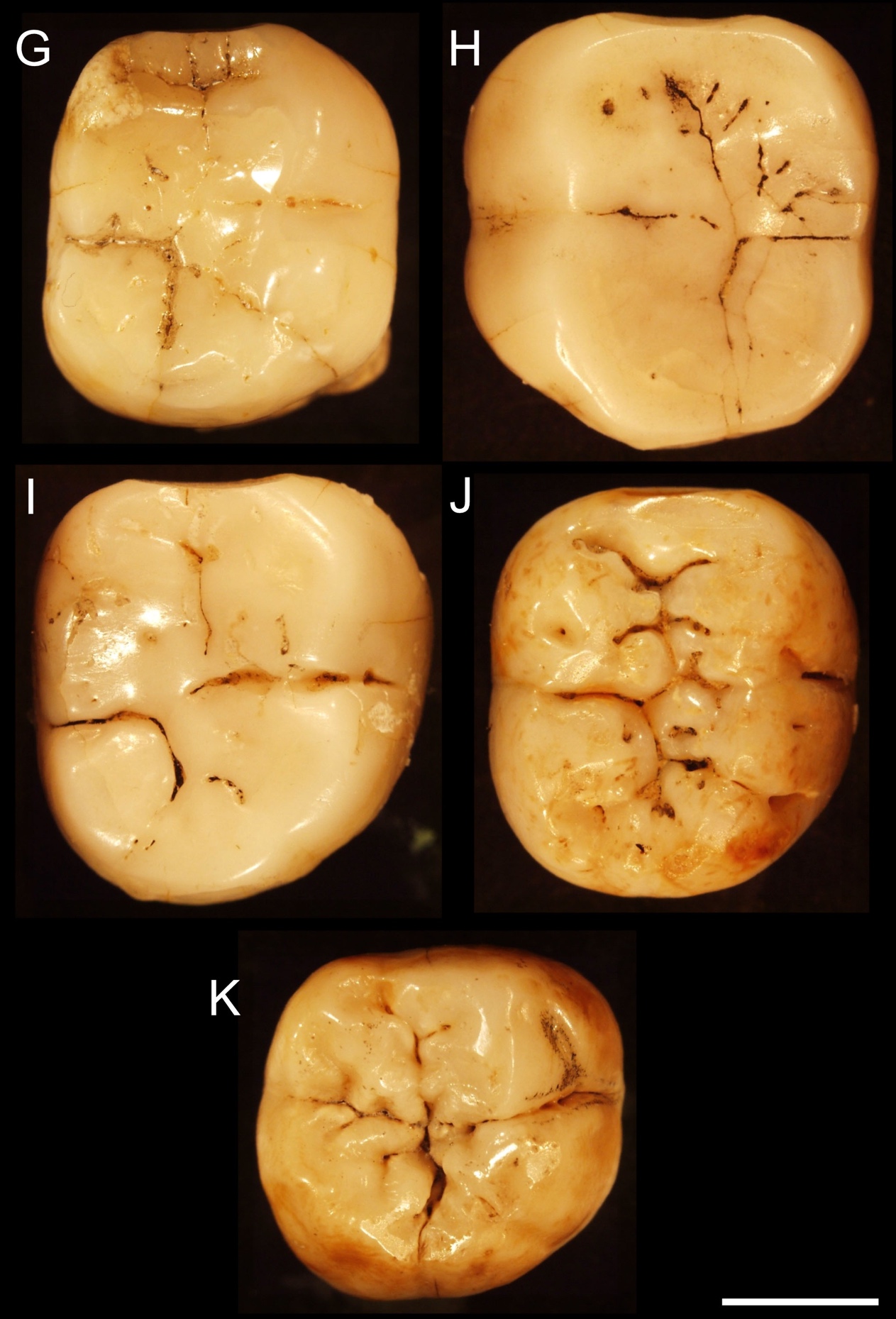


(A) 770 (type), (B) 771, (C) 772, (D) 796, (E) 799, (F) 804, (G) 805, (H) 806, (I) 807, (J) 808, (K) 816. Scale bar is equal to 5 mm.

**Figure C. Sangiran dental material attributed to *Homo erectus* or fossil *Pongo* by Grine and included in this study.**


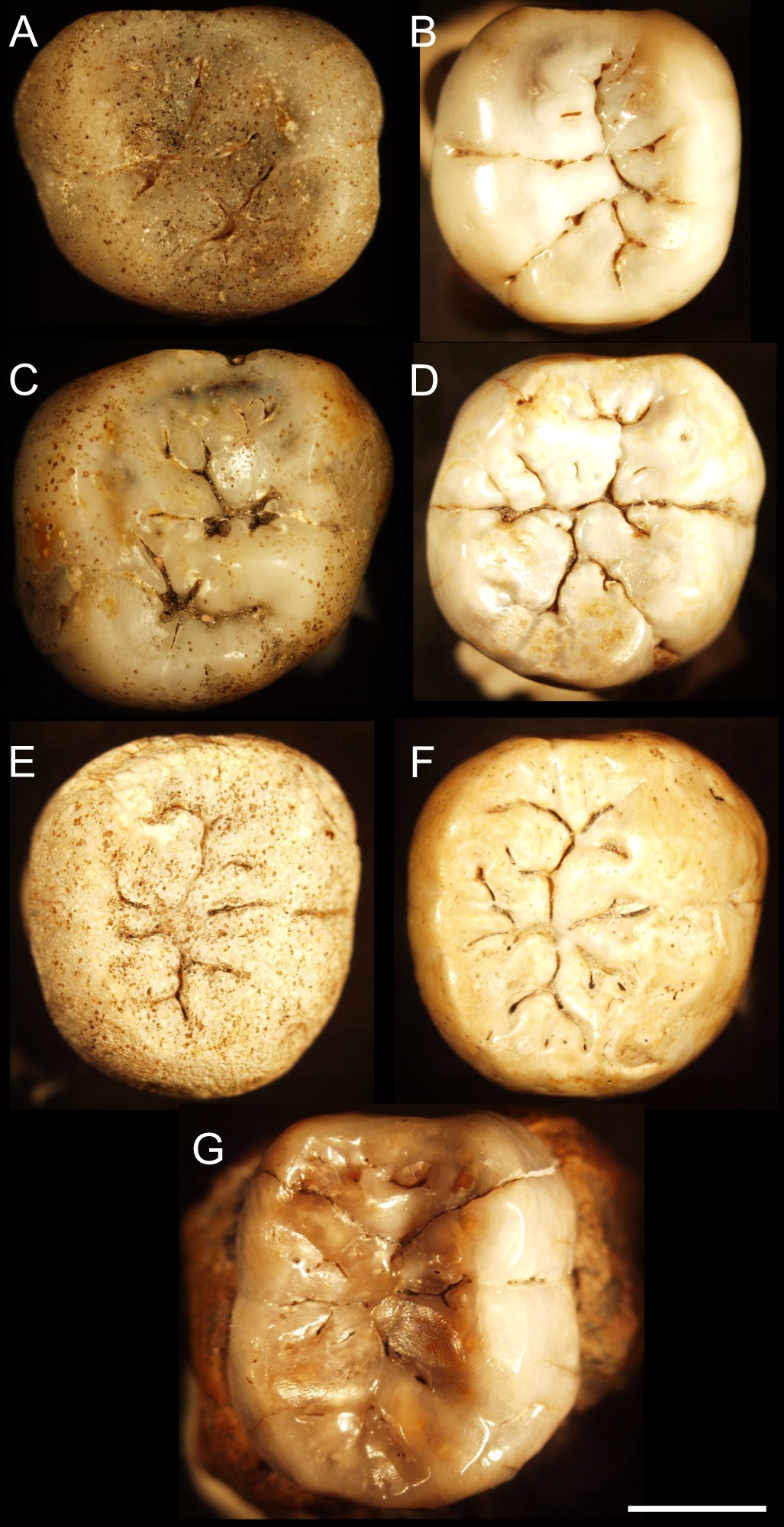


(A) S7-9, (B) S7-20, (C) S7-53, (D) S7-62, (E) S7-64, (F) S7-65, (G) S7-76. Scale bar is equal to 5 mm. See ref. [5] for a description of the Sangiran dome collection, including metrics for all teeth.

**Additional References**

101. Schlosser M. Die fossilen Saugetiere Chinas nebst einer Odonthographie der recenten Antilopen. Abh bayer Akad Wiss Math-phys. K1. 1903; 22: 20-21.

102. Liu W, Wu X. The hominid fossils from China contemporaneous with the Neanderthals and some related studies. In: Condemi S, Weniger G-C, editors. Continuity and discontinuity in the peopling of Europe: one hundred fifty years of Neanderthal study. Dordrecht: Springer; 2011; pp. 77-93. DOI 10.1007/978-94-007-0492-3_7

103. Institute of Vertebrate Paleontology and Paleoanthropology, eds. Atlas of primitive man in China. Beijing: Science Press; 1980.

104. Wu R, Dong X. *Homo erectus* in China. In: Wu R, Olsen JW, editors. Paleoanthropology and Paleolithic archaeology in the People’s Republic of China. Orlando: Academic Press; 1985. pp. 79-89.

105. Limbrey S. China. In: Oakley KP, Campbell BG, Molleson TI, editors. Catalogue of fossil hominids part III: Americas, Asia, Australasia. London: British Museum (Natural History); 1975. pp. 49-87.

106. Liu W, Si X. The human teeth discovered in Dadong, Panxian County, Guizhou Province. Acta Anthropol Sinica 1997; 16: 193-200.

107. Shen G, Wang W, Wang Q, Pan Y. U-series dating of hominid site Ganqian cave at tubo, Liujiang, Guangxi in south China. Acta Anthropol Sinica 2001; 20: 238-244.

108. Liu W, Wu X, Pei S, Wu X. A preliminary report on Huanglong Cave: a late Pleistocene human fossil site in Hubei Province, China. Quat Int. 2010; 211: 29-41.

109. Wang W, Huang C, Xie S, Yan C. Late Pleistocene hominin teeth from the Jimuyan Cave, Pingle County, Guangxi, South China. Quat Sci. 2011; 31: 699–704.

110. Gao J. *Australopithecus* teeth associated with *Gigantopithecus*. Vetrebrata PalAsiatica 1975; 13: 81-88.

111. Bae CJ, Wang W, Zhao J, Huang S, Tian F, Shen G. Modern human teeth from Late Pleistocene Luna Cave (Guangxi, China). Quat Int. 2014; 354: 169-183.

112. Wu X, Wu M. Early *Homo sapiens* in China. In: Wu R, Olsen JW, editors. Paleoanthropology and Paleolithic archaeology in the People’s Republic of China. Orlando: Academic Press; 1985. pp. 91-106.

113. Wu M. New discoveries of human fossil in Tongzi, Guizhou. Acta Anthropol Sinica 1984; 3: 195-201.

114. Liu W, Jin C-Z, Zhang Y-Q, Cai Y-J, Xing S, Wu X-J. et al. Human remains from Zhirendong, South China, and modern human emergence in East Asia. Proc Natl Acad Sci U S A 2010; 107: 19201-19206.

115. Bergh GD van den, Kaifu Y, Kurniawan I, Kono RT, Brumm A, Setiyabudi E, et al. *Homo floresiensis*-like fossils from the early Middle Pleistocene of Flores. Nature 2016; 534: 245-248.

116. Demeter F, Sayavongkhamdy T, Patole-Edoumba E, Coupey A-S, Bacon AM, Vos J de, et al. Tam Hang rockshelter: preliminary study of a prehistoric site in Northern Laos. Asian Perspectives 2009; 48: 291-308.

117. Storm P, Aziz F, Vos j de, Kosasih D, Baskoro S, Ngaliman, et al. Late Pleistocene *Homo sapiens* in a tropical rainforest fauna in East Java. J Hum Evol. 2005; 49: 536**-**545.

118. Dubois E. Voorloopig bericht omtrent het onderzoek naar de Pleistocene en tertiaire Vertebraten-Fauna van Sumatra en Java, gedurende het jaar 1890. Natuurk Tijdschr Ned Ind 1891; 51: 93-100.

119. Tougard C, Chaimanee Y, Suteethorn V, Triamwichanon S, Jaeger J-J. Discovery of a *Homo* sp. tooth associated with a mammalian cave fauna of Late Middle Pleistocene age, Northern Thailand. J Hum Evol. 1998; 35: 47-54.

120. Kha LT. First remarks on the Quaternary fossil fauna of northern Vietnam. Vietnam Stud. 1976; 46: 107-126.

121. Olsen JW, Ciochon RL. A review of evidence for postulated Middle Pleistocene occupations in Viet Nam. J Hum Evol. 1990; 19: 761-788.

122. Ciochon RL, Olsen JW, James J. Other origins: The search for the giant ape in human prehistory. 1^st^ ed. New York: Bantam Books; 1990.
